# Single-cell and spatial dissection of precancerous lesions underlying the initiation process of oral squamous cell carcinoma

**DOI:** 10.1038/s41421-023-00532-4

**Published:** 2023-03-13

**Authors:** Lulu Sun, Xindan Kang, Chong Wang, Rui Wang, Guizhu Yang, Wen Jiang, Qi Wu, Yujue Wang, Yaping Wu, Jiamin Gao, Lan Chen, Jie Zhang, Zhen Tian, Guopei Zhu, Shuyang Sun

**Affiliations:** 1 Department of Oral and Maxillofacial-Head Neck Oncology, Shanghai Ninth People’s Hospital, Shanghai Jiao Tong University School of Medicine, Shanghai, China; 2 College of Stomatology, Shanghai Jiao Tong University, National Center for Stomatology, National Clinical Research Center for Oral Diseases, Shanghai Key Laboratory of Stomatology, Shanghai Research Institute of Stomatology, Shanghai, China; 3 Department of Oral and Maxillofacial-Head Neck Oncology, Division of Radiation Oncology, Shanghai Ninth People’s Hospital, Shanghai Jiao Tong University School of Medicine, Shanghai, China; 4 Department of Oral Pathology, Shanghai Ninth People’s Hospital, Shanghai Jiao Tong University School of Medicine, Shanghai, China

**Keywords:** Oral cancer, Cancer prevention, Transcriptomics

## Abstract

Precancerous lesions of the oral mucosa, especially those accompanied by moderate to severe dysplasia, contribute to the initiation of oral squamous cell carcinoma (OSCC). However, the cellular compositions and spatial organization of the precancerous stage and how these factors promote human OSCC initiation remain unclear. Here, we built a single-cell transcriptome atlas and a spatial transcriptome map after obtaining data from pairwise human oral mucosal biopsies of 9 individuals consisting of very early-stage OSCC, adjacent precancerous lesions with moderate to severe dysplasia, as well as a matched normal region. An altered epithelial gene-expression profile was identified which favored OSCC initiation. This observation was coupled with distinct fibroblast, monocytic, and regulatory T-cell subclusters involved in reshaping the microenvironment. In particular, a unique immune-inhibitory monocyte subtype and spatial-switching regulation of VEGF signaling were observed surrounding precancerous lesions, concertedly strengthening activities in promoting cancer initiation. Collectively, our work elucidated the cellular landscapes and roles of precancerous lesions underlying OSCC initiation, which is essential for understanding the entire OSCC initiation process and helps inform therapeutic strategies for cancer intervention.

## Introduction

Solid cancers account for ~90% of human malignancies, and their ontology is a complex multi-stage process. Precancerous lesions, which present prior to the onset of cancer, have been reported to contribute to cancer initiation^1^. Understanding the precise molecular and cellular mechanisms underlying the acquisition of aberrant phenotypes at precancerous and further carcinogenesis stage is essential for developing early diagnostic and preventative strategies^1^. However, it is difficult to capture fresh solid tumor samples containing aberrant precancerous lesions from visceral tissues. Previous studies have reported on micro-dissection of putative precancerous and cancer regions from paraffin-fixed clinical samples for inherent variation analysis, but these reports failed to unveil potential biomarkers or communications between malignant cells and tumor microenvironments underlying human cancer initiation^2^.

The oral cavity, which directly connects to the external environment, offers a unique opportunity for capturing the occurrence of precancerous lesions and subsequent tumor. Oral squamous cell carcinoma (OSCC), the most common oral carcinoma (accounting for ~90%), exhibits a certain correlation between its onset and the precancerous lesions of the oral mucosa^3,4^. Oral leukoplakia (OLK) is one of the most common precancerous lesions and has a rate of ~20% to undergo malignant transformation to OSCC, and an even higher risk when accompanied with the occurrence of moderate to severe dysplasia^5^. Thus, it is possible to capture and study OSCC initiation within or around the OLK region. Previous work has reported several factors, such as loss of heterozygosity (for example, in the 3p14 and 9p21 regions), as well as dysregulated EGFR and PI3K-AKT− mTOR signaling in OLK epithelium, all of which are related to OSCC initiation^6,7^. However, there has been little investigation into the entire cellular landscapes and spatial organizations at the precancerous stage of OSCC initiation.

Single-cell RNA sequencing (scRNA-seq) enables the dissection of cellular subpopulations and to explore specific characteristics and factors associated with disease development or therapeutic treatment, especially in full-blown cancers^8–12^. Recent advances in spatial transcriptomics (ST) have enabled the acquisition of spatial organization information and transcriptome data simultaneously, thereby generating a comprehensive, spatial-temporal view of gene expression across a specific tissue or over the course of disease development^13,14^.

Herein, we combined scRNA-seq and ST techniques to profile pairwise biopsies, which simultaneously contained early OSCCs at the T1 stage, adjacent OLK with moderate to severe dysplasia, and a matched normal region. The integrated single-cell transcriptome combined with the spatial organization information enabled the comprehensive study of cell populations and transcriptional distributions underlying OSCC initiation. We uncovered a set of initiation-associated genes in the epithelium, alongside initiation-associated fibroblasts and immune subclusters, with their synergistic efforts promoting carcinogenesis. Of note, an immune-inhibitory monocyte subtype and specific spatial-switching communications were identified to be involved in reshaping the microenvironment of OLK. Together, our data and results serve as a reference for further OSCC studies and the development of potential strategies for cancer intervention.

## Results

### scRNA-seq and ST profiling of early OSCC biopsies obtained from normal, OLK, and tumor regions

**Fig. 1:**
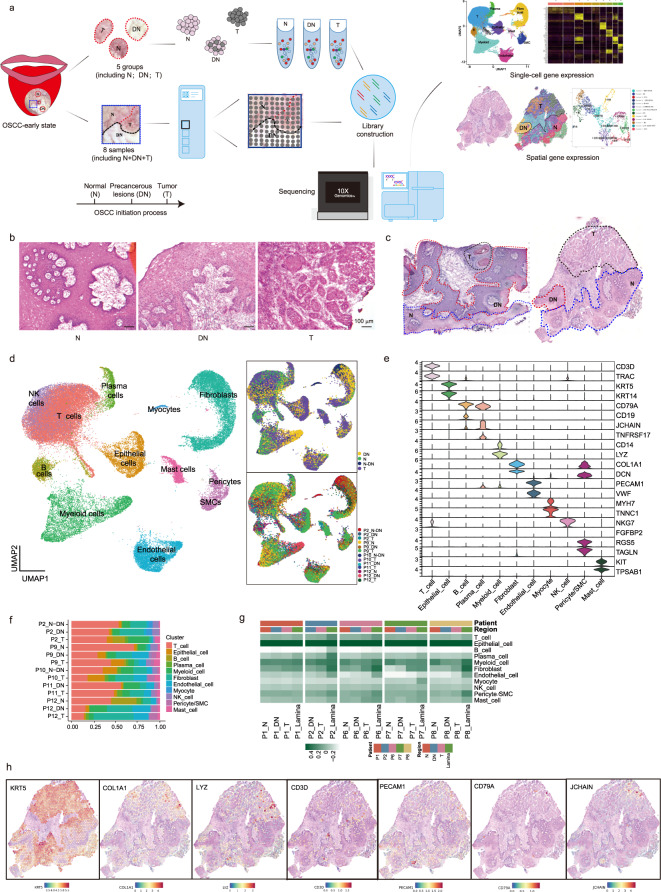
A single-cell and ST profiling of pairwise oral mucosal biopsies, comprising early OSCCs, adjacent precancerous lesions, and matched normal regions. **a** Schematic diagram illustrating the workflow of human oral mucosal biopsies processing for scRNA-seq and ST analyses. N normal, DN dysplasia, T tumor. **b** Representative H&E staining of tissue samples biopsied for scRNA-seq at the distinct stage during OSCC initiation. Scale bar: 100 μm. **c** H&E images of ST samples. Initiation stages were indicated at different spatial locations under the guidance of a clinical pathologist. **d** UMAP representation of single cells profiled in the presenting work colored by major cell types (left), tumor initiation stages (right top) and individuals (right, bottom). **e** Expression levels of diverse marker genes by annotated cell types. **f** Bar plots showing the proportion of cell types included in each individual for scRNA-seq. **g** Heatmap displaying enrichment of cell types in ST samples which were annotated in scRNA-seq. Note that the labeled initiation stages of ST samples were carefully divided under the guidance of a clinical pathologist. **h** ST feature plots of P1 showing expression of marker genes by annotated cell types.

To investigate how the cellular compositions and spatial organization of OLK and its participation in human OSCC initiation, we chose tissue samples simultaneously containing early OSCCs at the T1 stage (T), OLK with moderate to severe dysplasia being adjacent (DN), and matched normal region (N). In general, 10 tissue samples from 9 individuals met the criteria, including 5 pairs biopsies for scRNA-seq (P2, P9-P12; here P denotes patient ID) and the other 5 for ST analyses (P1, P2, P6–P8) (Supplementary Table S1; see “Materials and methods”). For scRNA-seq, biopsies of N, DN, and T regions from each individual were processed separately, while N, DN, and T lesions of each individual were included in a ST feature plot. The scRNA-seq data were generated after cell number assessment and pathology confirmation (see “Materials and methods”; Fig. 1a, b and Supplementary Tables S1 and S2). The number of dissociated single cells in the N region of P2 as well as the N and DN regions of P10 was insufficient for conducting scRNA-seq; accordingly, single live cells from the N regions were mixed with some cells from DN for scRNA-seq (Supplementary Table S2). For ST analyses, eight biopsies (P1–P8) were performed and five ST feature plots finally met the criteria (see “Materials and methods”; Fig. 1a and Supplementary Tables S1 and S3). The transcriptomic and H&E image data were provided and the histological regions were then labeled by the experts (Fig. 1c and Supplementary Fig. S1a, b).

After quality filtering and doublet removal, a total of 52,721 high-quality single cells were profiled by RNA-seq using the 10× Genomics Chromium Droplet platform. The mean sequencing depth was 43,905 reads per cell (Fig. 1d–f and Supplementary Table S2). Following gene-expression normalization and principal component analysis (PCA) in evaluating variably expressed genes, single cells were unsupervisedly clustered into 11 major clusters and annotated by cluster-specific marker genes (Fig. 1d–f). Most markers were classic and had been annotated in the previous studies: oral epithelial cells (*KRT5* and *KRT14*), endothelial cells (*PECAM* and *VWF*), fibroblasts (*COL1A1* and *DCN*), B/Plasma cells (*CD79A*, *CD19*, *JCHAIN*, and *TNFRSF17*), myeloid cells (*CD14* and *LYZ*), T cells (*CD3D* and *TRAC*), myocyte cells (*MYH7* and *TNNC1*), pericytes/SMCs (*RGS5* and *TAGLN*), and mast cells (*KIT* and *TPSAB1*) (Fig. 1d, e and Supplementary Tables S4, S6).

For ST analyses, a total of 26,773 tissue spots within these 8 sections were sequenced, with a median unique molecular identifiers (UMI) value of 17,853 per spot (Supplementary Fig. S1b, c and Table S3). The cell-type occupation and enrichment were then displayed in the heatmap by scoring spots of cell-type specific markers in N, DN, T, and lamina regions of each ST section (Fig. 1g and Supplementary Table S5). Lamina denotes connective tissues below the epithelium. Interestingly, epithelial cells were ranked first, followed by fibroblasts, myeloid cells and T cells in terms of ST data (Fig. 1g). Representative expression features of specific cell-type markers were also illustrated in P1 (Fig. 1h). Moreover, correlation heatmaps of major cell types at N, DN, and T regions of ST feature plots demonstrated that epithelial cells had an increased correlation with fibroblasts, myeloid and T cells at DN and T stages, indicating their potential communications during the OSCC initiation (Supplementary Fig. S1d and Table S5).

In the following analyses, cells in each cluster were further divided into subclusters and were respectively annotated with unique markers for evaluating their functions in OSCC initiation (Supplementary Fig. S1e).

### Correlation of high copy number variation (CNV), FRA, and mTORC1 signaling scores of epithelial cells with OSCC initiation

**Fig. 2:**
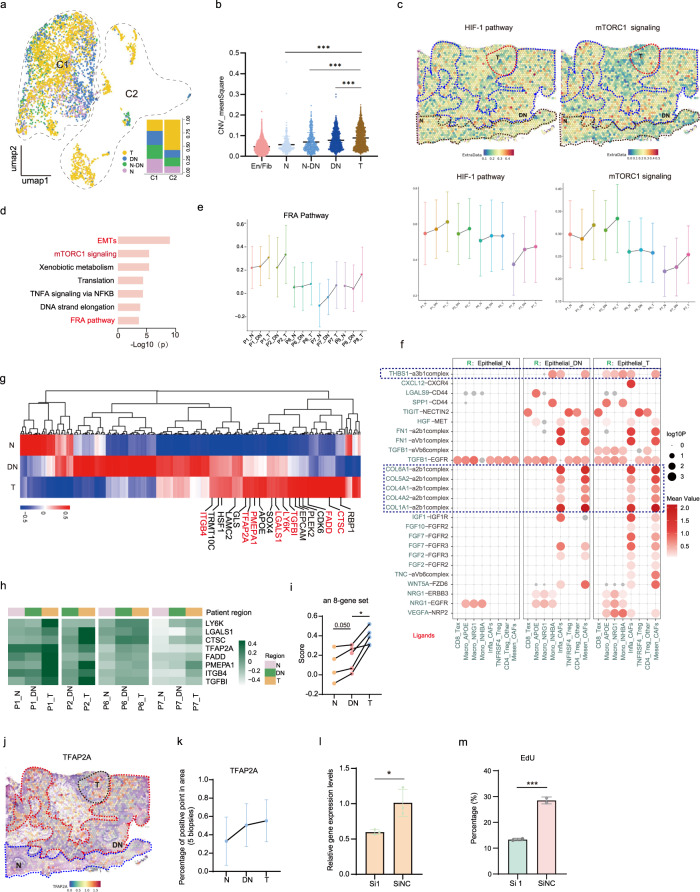
Identification of the initiation-associated epithelial markers and pathways. **a** UMAP plots showing epithelial cells of diverse stages. Their proportions in C1 and C2 clusters were circled in the map and the statistics were shown in bar plot. **b** Statistical results showing CNV-meanSquare of epithelial cells at different initiation stages. Endothelial cells and fibroblasts as a whole represented the baseline reference. Nonparametric unpaired *t-*tests were used to calculate the statistical significance. ^***^P < 0.001. **c** Characterization of OSCC initiation process with ST feature plots from P6 showing cancer-related pathways (Top). Changes of these pathways’ activities in patients of ST. Each dot indicated the median of the pathway activity in the corresponding N, DN or T region (Bottom). **d** Enriched GO functions of DEGs with gradual-increased expression among epithelial cells from N, DN, and T stages. FDR *q* value < 0.05. **e** Changes of FRA pathway activities along with tumor initiation process in ST. Each dot indicated the median of the pathway activity in the corresponding region. **f** Dotplots showing the significance (−log10 *P* value) and strength (mean value) of specific interactions between T, Myeloid, and cancer-associated fibroblast (CAF) cells with epithelial cells at N, DN, and T stages. Significant mean and significance (*P* < 0.05) were calculated based on the interaction and the normalized cell matrix was achieved by Seurat Normalization. **g** Heatmap of DEGs between epithelial cells of diverse initiation stages. The eight genes confirmed by ST were marked red. **h** Heatmap for statistical analyses of 8 initiation-associated genes expression in ST feature plots (each row shared a color scale, while different columns did not). **i** Statistical analysis of the eight initiation-associated genes expression in five ST feature plots. The dot plots display statistical score values for theses eight-gene sets in each region of tissue sections. A two-tailed paired Student’s *t-*test for the *P* values. ^*^P < 0.05. **j** Spatial feature plots of TFAP2A in tissue sections of P6. **k** Statistical results showing proportions of TFAP2A positive points in different tissue regions by ST analyses. **l** The gene-expression levels of TFAP2A in siTFAP2A and siNC group. Data were presented as the mean ± s.d. NC control group. **m** Quantification of EdU-positive cell proportions in siTFAP2A groups. Four representative pictures of each group were used for quantification. A two-tailed unpaired Student’s *t*-test for the *P* values in **l**, **m**. ^*^P < 0.05; ^***^P < 0.001.

After re-clustering entire epithelial cells, 4180 cells were separated into subclusters based on their expression states (Fig. 2a). Clustering results indicated that the majority of subclusters resided closer to each other (C1), with a small fraction of subclusters distributed dispersively (C2), and most of cells in C2 were from T regions (Fig. 2a). As biopsies in our study represented a similar continuous progression from OLK to early OSCC, transcriptome characteristics could be similar, that is, the heterogeneity between patients had not been fully manifested.

To investigate whether variations could separate malignant cells from non-malignant cells based on normal karyotypes, we next inferred large-scale chromosomal CNVs in each epithelial cell. The statistical scores of CNV (mean square of alterations per cell, including both CNV gain and loss) showed that epithelial cells in DN and T regions were gradually enriched in CNVs (Fig. 2b). Several cells in the normal group presented CNV variation, possibly due to the nature of the algorithm, sample collection operation, or later treatment on cells; and these cells were excluded from following analyses. Representative CNV plots of P2 and P9 illustrated that a concordant variation trend was observed in similar chromosomal locations among N, DN, and T regions of an individual (Supplementary Fig. S2a). This pattern indicated a common lineage during the epithelial malignant transformation from N, DN, then to T stages, yet was unlikely due to the adjacent locations of the biopsies^15^. In addition, we identified recurrent CNVs in regions of well-recognized cancer-driving genes (*EGFR*, *MTOR*, *CCND1*, and *MYC*) or tumor suppressor gene (*RB1*), with a relatively higher variation in the DN and T stages (Supplementary Fig. S2b). The gradually enhanced CNV burden of paired biopsies also demonstrated the reliability and representatives of the sampling in the scRNA-seq experiment.

Many classical pathways and markers are essential in cancer formation, including glycolysis, hypoxia, and DNA mismatch repair^16,17^. Indeed, we confirmed an overall abundance trend of these pathways in DN and T regions of our spatial feature plots, though the tumor heterogeneity and surrounding stroma cells may interfere with the pathway score (Fig. 2c and Supplementary Fig. S2c, d). Specific to OSCCs, mTORC1 signaling, P63, and some stem cell markers, such as BMI and CD44, were reported as highly prevalent molecular signatures underlying OSCC pathogenesis^18–20^.

The gene ontology (GO) analysis of differently expressed genes (DEGs) with increased expression trend in epithelial cells from N, DN, and T stages indicated a gradual enrichment of EMT, mTORC1, and FRA pathways during the OSCC initiation process (from N, DN to T group) (Fig. 2d). Consistently, scoring plots of ST data unveiled a similar variation trend in the FRA and mTORC1 pathways (Fig. 2e, c). In addition, CellphoneDB analysis showed few interactions between the stroma and epithelial cells at N stages. However, from the DN to T stage, we observed frequent communication between epithelial cells and the surrounding stromal cells, indicating a dramatic change in expression profiles of epithelial cells and interactions at the DN stage (Fig. 2f).

### Identification of OSCC initiation-associated epithelial markers that were involved in regulating the OSCC initiation process

In order to identify potentially essential epithelial genes with gradual upregulation during OSCC initiation, which we hereafter termed as “initiation-associated” genes (Fig. 2g). ST maps were developed as intuitive sources of confirmation. Accordingly, eight initiation-associated genes (an eight-gene set) were identified by scRNA-seq and confirmed using ST maps, including: *CTSC*, *FADD*, *ITGB4*, *LGALS1*, *LY6K*, *PMEPA1*, *TFAP2A*, and *TGFβI* (Fig. 2h, i and Supplementary Fig. S2e). The chromosomal regions of some initiation-associated genes were found to be recurrently amplified in these patients, potentially promoting higher expression in DN and T stages (Supplementary Fig. S2b). Notably, *TFAP2A* encodes a transcription factor (TF) in the FRA pathway, *LY6K* encodes a lymphocyte antigen, *CTSC* is a member in the cysteine cathepsin family, and *LGALS1* encodes a galectin-regulating cell–cell/matrix interactions (Fig. 2h). These eight genes were highly expressed in tumor tissues of the TCGA head and neck squamous cell carcinoma (HNSCC) dataset, and that higher expression of this eight-gene set was significantly correlated with poor prognosis (Supplementary Fig. S2f, g, GEPIA 2 website, see “Materials and methods”).

Two of these eight potential biomarkers were further confirmed. The FRA/AP pathway, as well as the key transcriptional factor in this pathway, TFAP2A, were gradually enriched in OSCC initiation. Previous work on nasopharyngeal carcinoma also reported the involvement of TFAP2A in the early occurrence of SCC^21^. The gradually increased expression trend of TFAP2A in epithelial cells alongside OSCC initiation was verified by ST feature plots and histological staining (Fig. 2j, k and Supplementary Fig. S3a). Upon knocking down of *TFAP2A* in OLK organoids in vitro, proliferation was slowed, with decreased expression of other initiation-associated genes (Fig. 2l, m and Supplementary Fig. S3b). This likely reflected the potential role of TFAP2A in regulating OSCC initiation.

A second gene, *LY6K*, belongs to a lymphocyte antigen. It is highly expressed in tumor regions of lung SCC, esophageal cancers, and HNSCC^22,23^ (Supplementary Fig. S3c). Its role in OSCC initiation requires further study. Our ST data and histological staining verified a gradual increased expression in epithelial cells corresponding with OSCC initiation (Supplementary Fig. S3d, e). Moreover, in vitro data demonstrated that OSCC organoids exhibited higher expression of *LY6K* compared to organoids from DN biopsies (Supplementary Fig. S3f). Knocking down of *LY6K* in OLK organoids was corresponded with down-regulation of the expression of several proliferation and initiation-associated genes, including *CD44*, *CCND1*, *PMEPA1*, and *FOSL1*. This result indicated its potential role in influencing of OSCC initiation (Supplementary Fig. S3g, h). While TFAP2A and LY6K are promising for the identification or prevention of early SCC at the DN stage, more work is required to further evaluate their clinical values in OSCC initiation.

### The Mesen_CAF subcluster dominated OSCC initiation and was spatially closer to the epithelium when compared with Infla_CAF

**Fig. 3:**
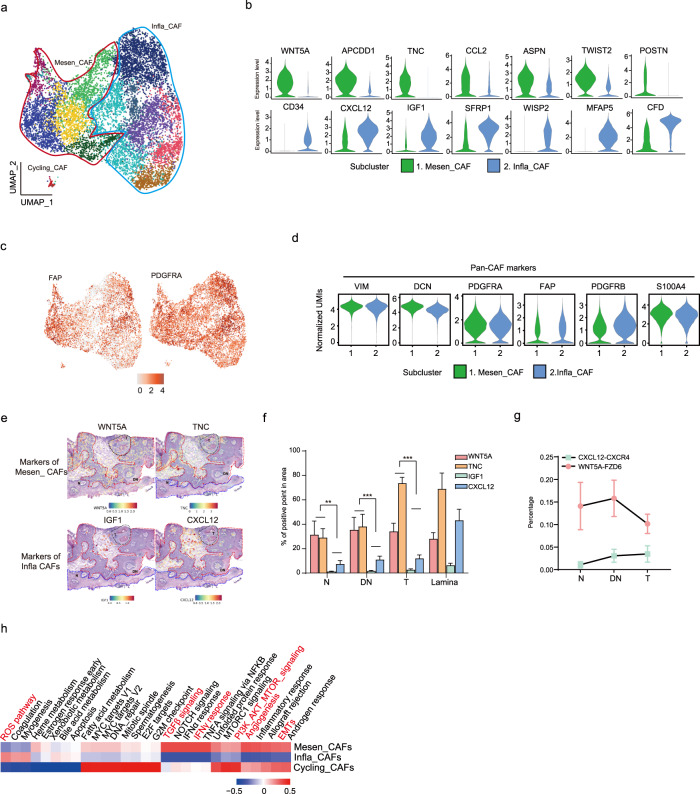
Mesen_CAF was prominent in OSCC initiation. **a** UMAP plots showing the subpopulations of fibroblasts. **b** Violin plot showing seven representative marker genes expressions of Mesen_CAFs and Infla_CAFs. **c** UMAP feature plots showing expression distribution of cancer-associated fibroblast cell markers FAP and PDGFRA in single fibroblast cells. **d** Violin plot showing gene-expression levels of pan-CAF markers in two fibroblasts’ subpopulations. **e** Spatial feature plots of Mesen_CAFs markers (WNT5A and TNC) and Infla_CAFs markers (IGF1 and CXCL12) in tissue sections of P6. **f** Statistical results showing proportions of (WNT5A, TNC, IGF1 and CXCL12) positive points in different tissue regions by ST analyses. A two-tailed unpaired Student’s *t-*test for the *P* values. ^**^P < 0.01. ^***^P < 0.001. **g** Statistics for scoring the positive points of CXCL12–CXCR4 and WNT5a–FZD6 interactions in 5 representative spatial feature plots. **h** Enriched Hallmark gene sets in Mesen_CAFs and Infla_CAFs with QuSAGE.

During cancer initiation, epithelial cells undergo malignant alterations. The microenvironment surrounding epithelial cells imposes selective pressure on cancer initiation^24^. We began by investigating fibroblast subpopulations. Based on our data and prior studies, fibroblasts were grouped into three major subpopulations: Mesen_CAF, Infla_CAF, and Cycling_CAF^25,26^ (Fig. 3a and Supplementary Fig. S4a, b). The Mesen_CAF subcluster had highly accumulated extracellular matrix (ECM) genes (*ASPN, POSTN,* and *TNC*) and mesenchymal-promoting TF (*TWIST2*), resembling reported myo-cafs (mCAFs)^25^ (Fig. 3b and Supplementary Fig. S4b). However, Mesen_CAF was simultaneously enriched in growth factors, including the non-classical WNT pathway (*APCDD1*, *PMEPA1* and *WNT5A*) and chemokine markers (*CCL2* and *IL1R1*) (Fig. 3b and Supplementary Fig. S4a, b, d). On the other hand, the Infla_CAF subcluster was enriched in the classical stromal-cell-derived factor *CXCL12*, and presented enhanced expression of hormone and metabolism-related markers (*IGF1* and *CFD*) (Fig. 3b and Supplementary Fig. S4a, b, d). The Cycling_CAF occupied a small population and was enriched in *MKI67*, *CDK1* and *UBE2C* genes, all of which were all related to cell proliferation. Importantly, these subclusters exhibited strong expression of classical CAF markers: *DCN*, *FAP*, *PDGFR-α/β*, *S100A4*, and *VIM*^27^ (Fig. 3c, d).

ST feature plots and the corresponding statistical analyses of marker staining area size revealed that Mesen_CAF markers were expressed with over 3-fold increased abundance in area than Infla_CAFs during OSCC initiation, with a gradually increasing trend (Fig. 3e–g). Mesen_CAF was widely distributed throughout epithelial regions, while Infla_CAF was more prevalent in the lamina region, suggesting the involvement of Mesen_CAF in epithelial cell carcinogenesis (Fig. 3e, f and Supplementary Fig. S4c). Indeed, secreted growth factors, such as FGFs, TNC, and WNT5A, started interaction with epithelial cells from the DN stage, likely contributing to epithelial proliferation (Fig. 2f). Gene set enrichment analysis using GO terms within these two fibroblast subclusters revealed that Mesen_CAF was enriched in TGFβ, EMT, angiogenesis, and PI3K-AKT-mTOR pathways. These pathways have previously been reported to be essential for cancer development, implying their potential roles in shaping the microenvironment towards OSCC initiation^17^ (Fig. 3h). In addition, some immune cells were proximal to Mesen_CAFs in epithelial regions (Supplementary Fig. S4e). CellphoneDB analysis inferred cellular communications between these cells, such as CSF1–CSF1R, TNC–a4b1 complex, and TGFβ–TGFβR interactions, potentially participating in immune cell recruitment and activation, as well as promoting Mesen_CAF collagen formation or mesenchymal functions during cancer initiation (Supplementary Fig. S4f). Taken together, both scRNA-seq and ST data revealed the functions of fibroblast subclusters in carcinogenesis.

### The Mono_INHBA subcluster was enriched in OLK and frequently interacted with CAFs, exhausted T, and epithelial cells at DN stage

**Fig. 4:**
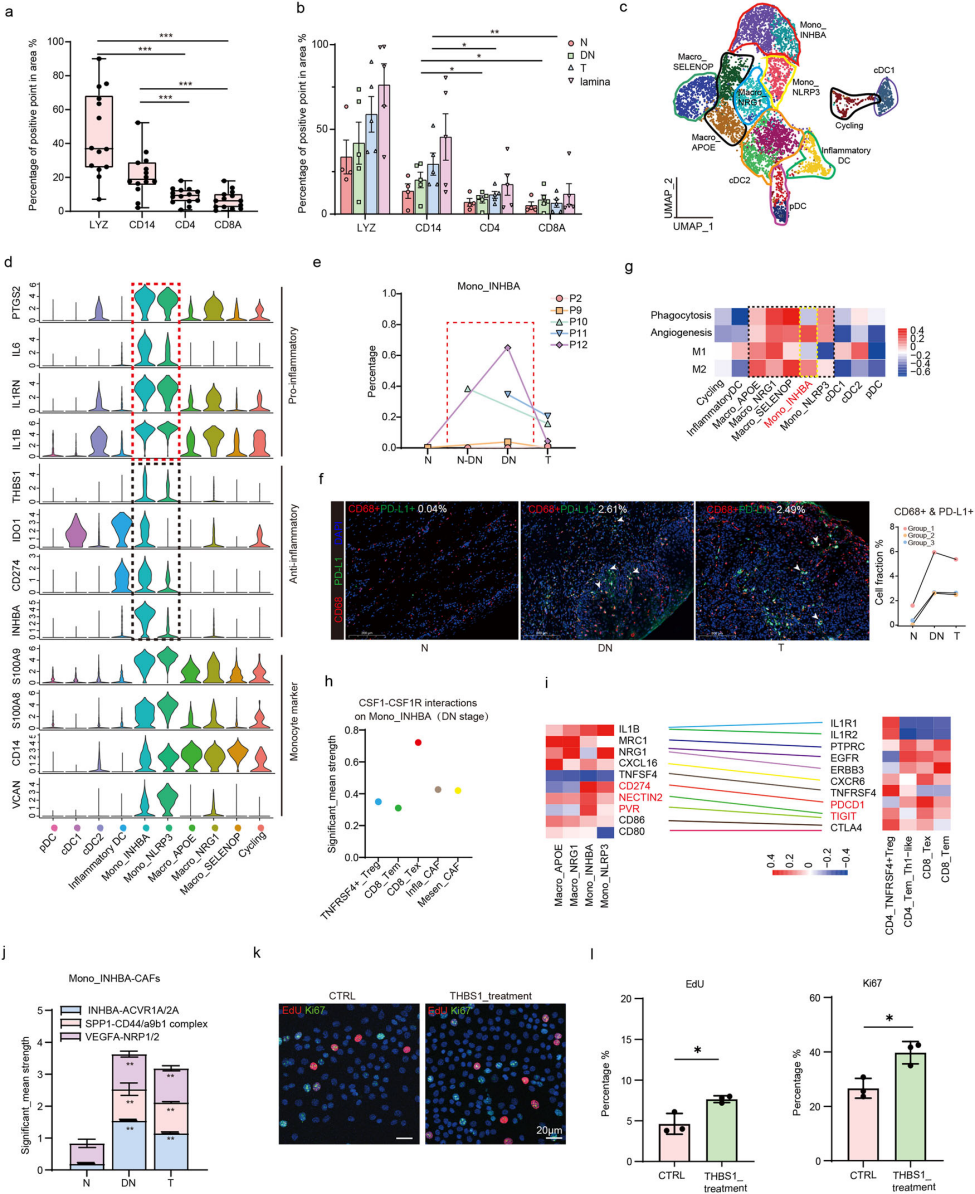
Mono_INHBA was enriched in DN stage and interacted frequently with other stromal cells. **a**, **b** Statistics showing percentage of positive points for LYZ, CD14, CD4, and CD8A in whole regions (including N, DN, T, and lamina region) (**a**) and separate regions (**b**) of the five representative spatial feature plots. A two-tailed paired Student’s *t-*test for the *P* values. ^*^P < 0.05, ^**^P < 0.01, ^***^P < 0.001. **c** UMAP representation of single myeloid cells colored by subclusters. Those irregular markers represent different identified cell types. **d** Violin plots showing expression levels of selected inflammation-associated genes and monocyte markers across different myeloid cell types from scRNA-seq data. **e** The line charts showing changes of Mono_INHBA cell proportions in each individual from N, DN to T stages. **f** mIHC staining of CD68 and PD-L1 in one patient of diverse initiation stages. Scale bars: 200 μm. Note that the statistic quantification was the average results of several representative pictures (left). The quantitation of CD68^+^ & PD-L1^+^ cell fractions in each group were provided (right). (*n* = 3 groups). **g** Enriched M1/M2/angiogenesis/phagocytosis pathways in different myeloid cell types done with QuSAGE. **h** CSF1–CSF1R interactions on Mono**_**INHBA in DN stage. Significant mean and significance (*P* < 0.05) were calculated based on the interaction and the normalized cell matrix achieved by Seurat Normalization. **i** Z-scored mean log expression heatmap of lymphatic ligands in monocytic cells (left) and receptors in T subclusters (right) from scRNA-seq data. Colored lines connect matching ligands for receptor. **j** Bar plots showing the significant_mean strength of representative interactions between Mono_INHBA and CAFs in N, DN, and T stages of OSCC initiation. Two-sided Wilcoxon rank-sum tests were used to calculate the statistical significance. ^**^P < 0.01. **k** Representative images of EdU and Ki67 staining in OLK organoids upon THBS1 treatment. Scale bars: 20 μm. **l** Quantification of EdU (left)/Ki67(right)-positive cell proportions in OLK organoids with THBS1 treatment. Three representative pictures of each group were used for quantification. A two-tailed unpaired Student’s *t-*test for the *P* values. ^*^P < 0.05.

Myeloid and T cells are key immune cells of the cancer ecosystem, both of which contribute to cancer formation through direct or indirect mechanisms^2^. However, their impact on OSCC initiation and progression is not well characterized. With the spatial feature map, we first analyzed the distributions of immune cells. Quantitation results of marker staining area size revealed that LYZ (a myeloid cell marker) and CD14 (a monocytic cell marker) were more abundant than two T cells markers, CD4A and CD8, in both whole-tissue sections or specifically to DN and T regions of a section, tending to be “cold tumors” with immune tolerance (Fig. 4a, b)^28^.

Among myeloid clusters, DC cells were characterized by *HLA-DRs*^+^ and *CD14*^–^ and further classified with reported markers^29^ (Fig. 4c and Supplementary Fig. S5a–c). The remaining clusters were identified as monocytic cells based on high expression of *CD68* and *CD14* (Fig. 4c and Supplementary Fig. S5a–c). Mono_INHBA and Mono_NLRP3 were classical *CD14*^+^ *CD16*^–^ monocytes, highly expressing monocyte markers, including *S100A8*, *S100A9*, and *VCAN* (Fig. 4d and Supplementary Fig. S5b). Macrophages were identified based on the enrichment of *CD16*, *CD163*, and *MRC1* and were then classified into three subclusters based on the enrichment of specific markers, namely Macro_SELENOP, Macro_APOE, and Macro_NRG1 (Supplementary Fig. S5b, c).

Our scRNA-seq data illustrated that macrophages were the most prevalent myeloid cells, accounting for 34.4% (total macrophages: 2248; total myeloid cells: 6538), while monocytes accounted for 26.5% on average with high variability among individuals (Supplementary Fig. S5d and Table S4). We identified several TFs specific to monocytes and macrophages: *IRF1* and *NKX3* were enriched in monocytes, while *NR1H3* and *MAFB* were highly expressed in macrophages (Supplementary Fig. S5e). Both monocytes subclusters, Mono_INHBA and Mono_NLRP3 presented comparable enrichment in *IL1B* and *IL6*, which were all key regulators of pro-inflammation, angiogenesis, and transformation into M2 macrophages^30^ (Fig. 4d). In addition, Mono_NLRP3 was enriched in *FCN* and *NLRP3*, two inflammatory factors (Supplementary Fig. S5b). However, cells in the Mono_INHBA subset exhibited high expression of reported anti-inflammatory factors, including *CD274* (PD-L1), *IDO1*, *INHBA*, *SPP1*, and *THBS1*^29^ (Fig. 4d). The mixed phenotype of Mono_INHBA shared high similarity with reported monocytic myeloid-derived suppressor cells (M_MDSCs), the pathologically activated monocytes with potential immune-suppressive activity^31^. The inflammatory mediators expressed by M_MDSCs have been documented to stimulate the accumulation and acquisition of functional MDSCs^32^.

Statistical analyses of cell numbers in myeloid cell subclusters with scRNA-seq data revealed that average proportions of the Mono_INHBA subset were highest in the DN region. However, two individuals had lower populations of Mono_INHBA in total (Fig. 4e and Supplementary Fig. S5f). The enrichment of Mono_INHBA was further validated by multiplexed immunohistochemistry (mIHC) staining, with the highest cell fractions in the DN stage (Fig. 4f). Heatmap results reflected that Mono_INHBA was enriched in M2-like immune-inhibitory and angiogenesis activities (Fig. 4g). Moreover, we found that exhausted CD8^+^ T cells (CD8^+^ Tex) were essential for recruitment of Mono_INHBA in the DN stage. This was because Mono_INHBA was spatially closer to CD8^+^ Tex and they had the strongest interactions through a CSF1–CSF1R interacting pair (Fig. 4h and Supplementary Fig. S5g, h). In turn, Mono_INHBA interacted with CD8^+^ Tex through CD274–PDCD1 and PVR/NECTIN2–TIGIT in promoting immune tolerance at the DN region, which were consistent with the enrichment of an M2-like pathway (Fig. 4g, i). INHBA is a member of the TGFβ1 superfamily, and we observed a correlation in its expression levels to *WNT5A*, a Mesen_CAF marker from the scRNA-seq data (Supplementary Fig. S5i). CellPhoneDB analysis indicated that Mono_INHBA communicated with CAFs mediated by INHBA-ACVR_1A/2 A, SPP1-CD44, and VEGFA-NRP1/NRP2 ligand-receptor pairs, modulating its collagen formation and other mesenchymal activities^33^ (Fig. 4j). To address if Mono_INHBA was associated with epithelial malignancy, we examined its interactions with epithelial cells during OSCC initiation. Interestingly, significant communication with epithelial cells through THBS1–a3b1 complex and SPP1–CD44 was found from DN stage (Fig. 2f). We mimicked their interactions through the addition of THBS1 to OLK organoids in vitro. Remarkably, our results demonstrated that THBS1 promoted epithelial proliferation and expression of initiation-associated genes identified above (Fig. 4k, l and Supplementary Fig. S5j). All data implied that Mono_INHBA was recruited to the DN region and “primed” for cancer formation through frequent communication with the entire ecosystem, mostly involving in inhibition of inflammation, ECM organization, epithelial proliferation, and expression profile alteration. Additionally, our analysis revealed that heightened expression of INHBA resulted in poor survival using the TCGA_HNSCC cohort data, further supporting the roles of INHBA in OSCC development^31^ (Supplementary Fig. S5k). Collectively, our results suggested the possible role of Mono_INHBA in shaping the OLK microenvironment leading to cancer initiation.

### Cluster 2-Mesen_CAF, Macro_APOE/NRG1, and TNFRSF4 ^+^ Tregs were involved in the DN to T-stage transition

**Fig. 5:**
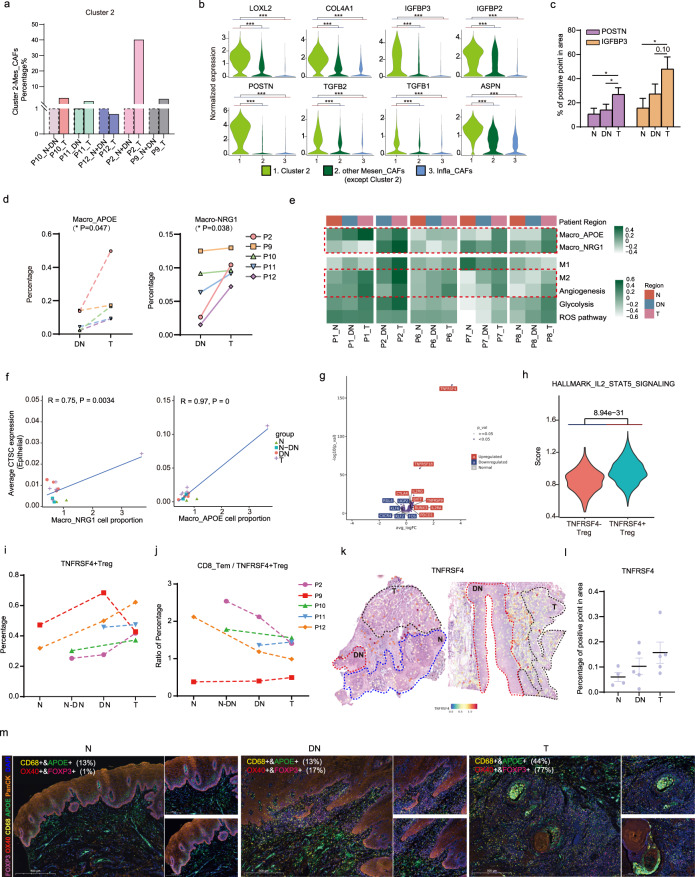
Cluster 2-Mesen_CAF, Macro_APOE/NRG1 and TNFRSF4^+^ Treg cells were increased from DN to T stage. **a** Cell proportions of Cluster 2-Mesen_CAF (Cluster 2-Mesen_CAF/CAF) in patients of T stage compared with N + DN stages by scRNA-seq data. **b** Violin plot displaying Cluster 2 marker gene expressions in Cluster 2-Mesen_CAF, other clusters in Mesen_CAF and Infla_CAF. Two-sided Wilcoxon rank-sum tests for analyzing the significance of their differences. ^***^P < 0.001. **c** Statistical results showing proportions of (*IGFBP3*, *POSTN*) positive points in different tissue regions by ST analyses. A two-tailed unpaired Student’s *t* test for *t*he *P* values. ^*^P < 0.05. **d** The line charts showing changes of (Macro_APOE/NRG1) cell proportions in each individual from DN to T stage. A two-tailed paired Student’s *t* test for the *P* values. ^*^P < 0.05. **e** Heatmap showing enrichment Macro_APOE/NRG1 and degrees of M1/M2/angiogenesis/glycolysis/ROS pathways by comparison of their marker genes expression levels in different distributions of ST samples. Each row shared a color scale, while different columns did not. **f** Pearson correlation plot showing significant positive correlation of Macro_APOE/NRG1 proportion to average CTSC expression in the epithelial cells. Cor.test() was used for conducting correlation and the statistical analyses. **g** Volcano plots showing strikingly DEGs between TNFRSF4^+^ Tregs and TNFRSF4^−^ Tregs. **h** Violin plots showing the metagene expression of the IL2-STAT5 signaling in TNFRSF4^+^ and TNFRSF4^−^ Tregs. *P* value was calculated by Wilcoxon test. **i** Cell proportions of TNFRSF4^+^ Treg cells in patients of diverse initiation stages by scRNA-seq data. **j** Proportions of cell number ratios of CD8_Tem cells to CD4_TNFRS4^+^ Treg cells in patients by scRNA-seq data. **k** ST feature plots of P1 and P2 showing expression of *TNFRSF4* in different distributions. **l** Statistical results showing proportions of TNFRSF4 positive points in different tissue regions by ST analyses (right). **m** mIHC staining of APOE, CD68, FOXP3, OX40 (TNFRSF4) and panCK in one patient of diverse initiation stages. Scale bars: 500 μm. The numbers in images indicated (CD68^+^ &APOE^+^ ; FOXP3^+^ &OX40^+^) double-positive cell proportions among corresponding CD68^+^ and FOXP3^+^ cells.

DN to T transition was the tipping point for carcinogenesis. To understand this process during OSCC initiation, we next explored trigger or constitutive elements underlying the transition^34^. As previously mentioned, Mesen_CAF dominated OSCC initiation process. Especially from the DN to T stage, proportions of one cluster (Cluster 2) of Mesen_CAFs in each individual were obviously increased in the T region (Fig. 5a and Supplementary Fig. S6a). Cells in Cluster 2 of Mesen_CAF were specifically enriched in mesenchymal progenitor and stromal activation markers, including *LOXL2*, *POSTN*, *TGFβ1*, and *TGFβ2*, which probably held higher power in proliferation and ECM organization functions among the Mesen_CAF (Fig. 5b and Supplementary Fig. S6b). Notably, markers of Cluster 2-Mesen_CAF were consistently enriched in tumor regions in ST feature plots and statistical data (Fig. 5c and Supplementary Fig. S6c).

In addition, we found that proportions of Macro_NRG1 and Macro_APOE were increased from the DN to T stage in each individual, and similar trends were observed in ST data (Fig. 5d, e). Cells in Macro_NRG1 were enriched in *NRG1*, *CLEC10A*, and *MRC1* (Supplementary Fig. S5b). Cells in Macro_APOE expressed a high level of identical M2-macropage markers, such as *CD163*, *C1QC*, and *TREM2* (Supplementary Fig. S5b, c). ScRNA-seq data showed that Macro_NRG1 and Macro_APOE were enriched in M2 as well as M1-like macrophage phenotype, together with ROS and angiogenesis pathways (Fig. 4g and Supplementary Fig. S6d, e). ST data revealed that M2-like macrophage and angiogenesis pathways exhibited an increase trend from DN to T stages, which indicated a joint effort of immune suppression of these immune cells (Fig. 5e). In addition, cellular proportions of these two subtypes were highly correlated with expression levels of epithelial *CTSC*, an initiation-associated gene that we identified before (Fig. 5f). This indicated that the gradual increase in expression of epithelial *CTSC* may be responsible for recruitment or differentiation of Macro_APOE/NRG1, especially at the DN to T stage when they shared similar trends. The possible trajectories in acquisition of Macro_APOE/NRG1 potentially from Macro_SELENOP, the tissue-resident-like macrophages, were predicted with the pseudotime and RNA velocity analyses (Supplementary Fig. S6f). Similar recruitment functions of CTSC have been previously reported in breast cancer^35^.

TNFRSF4^+^ Tregs represented another type of initiation-associated immune-inhibitory cells present throughout OSCC initiation, making up approximately 25% of CD4^+^ T cells in the scRNA-seq data (Supplementary Fig. S7a–g). *TNFRSF4(OX40)* was a significant DEGs between two *FOXP3*^+^ & *CD25*^+^ Treg subgroups (Fig. 5g and Supplementary Fig. S7h, i). Moreover, it was enriched in IL2-STAT5 signaling—a major driver of *FOXP3* expression as well as other inhibitory markers, such as *CTLA4* and *TNFRSF9*^36^ (Fig. 5h and Supplementary Fig. S8a). ST features and statistics confirmed the increasing trend of TNFRSF4^+^ Tregs, *TNFRSF4* expression, and occupation during OSCC initiation (Fig. 5i, k, l). Importantly, the ratio of CD8_Tem to TNFRSF4^+^ Treg cells remained stable or gradually decreased from the N to T stage, implying a possible decline trend of T-cell cytotoxicity during OSCC initiation (Fig. 5j). This suggested that TNFRSF4^+^ Tregs contributed to immune tolerance during OSCC initiation. Importantly, mIHC staining of APOE, CD68, FOXP3, and OX40, in early OSCC samples further verified the involvement of these immune cells during the DN to T-stage transition (Fig. 5m and Supplementary Fig. S8b).

Together, this study revealed the critical roles of CAF and immune cell subclusters in reshaping the microenvironment from the DN to T stage underlying OSCC initiation. Interestingly, high expression of signature genes was similarly reported in TCGA_HNSCC cohort and another recent study^37^ (Supplementary Fig. S8c). Moreover, these three subclusters exhibited high correlation with each other in TCGA_HNSCC cohort, indicating concerted roles in OSCC development (Supplementary Fig. S8d).

### Spatial-switching regulation of VEGF signaling surrounding the DN stage

**Fig. 6:**
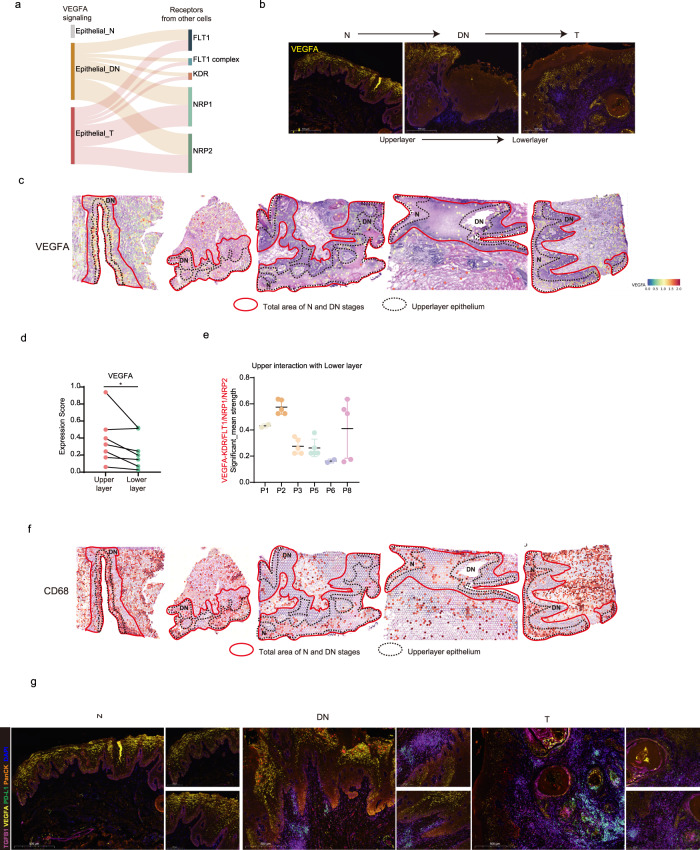
Spatial distribution of VEGF signaling during cancer initiation. **a** Alluvial plot showing selected VEGFA–NRP1/NRP2/KDR/FLT1 interactions between different initiation stages of epithelial cells and other cell subclusters. **b** mIHC staining of VEGFA in one patient of diverse initiation stages. Scale bars: 500 μm. **c** ST feature plots showing the expression of VEGFA in different distributions. Red solid lines circled total epithelium region of N and DN stages; black dotted lines indicated the upper layer of epithelium, mainly composed of differentiated keratinocytes. **d** Quantitative scores of expression levels of VEGFA in upper and lower layer of the corresponding ST sections. A two-tailed paired Student’s *t-*test for the *P* values. ^*^P < 0.05. **e** Scores of VEGFA-NRP1/NRP2/KDR/FLT1 interactions between the upper layer and lower layer of epithelium in diverse ST sections. Significant mean and significance (*P* < 0.05) were calculated based on the interaction and the normalized cell matrix achieved by Seurat Normalization. **f** ST feature plots showing the expression of CD68 in different distributions. Red solid lines circled total epithelium region of N and DN stages; black dotted lines indicated the upper layer of epithelium, mainly composed of differentiated keratinocytes. **g** mIHC staining of VEGFA, PD-L1, TGFβ1, and PanCK in one patient of diverse initiation stages. Scale bars: 500 μm.

Based on the above analyses of scRNA-seq and ST transcriptome data, we discovered that distinct stroma cell subclusters were involved in OSCC initiation. Some critical signaling pathways were found to contribute to specific functions of these cell subclusters. Some essential classic cancer hallmarks for cancer development were enriched during OSCC initiation, including VEGFA and TGFβ signaling, which were expressed in most cell types from scRNA-seq data (Supplementary Fig. S9a–d). For example, the above analyses demonstrated that cells in Cluster 2 of Mesen_CAF were enriched in *TGFβ1*. *INHBA*, the marker of Mono_INHBA, was also a family of TGFβ. Moreover, *VEGFA* was highly expressed in monocytes. After summarization of the VEGFA and TGFβ1 interactions inferred by CellphoneDB, we found that epithelium-secreted VEGFA interacted with receptors on stroma cells since DN stage (Fig. 6a). In addition, TGFβ1–TGFβR1/2/3 communications were gradually increased from the N to T stage (Supplementary Fig. S9d). These data indicated that VEGFA and TGFβ1 signalings were not only the hallmarks of full-blown cancer, but also promoted cancer initiation.

From VEGFA staining of the OSCC initiation tissues, we observed spatial-switching expression of VEGFA in the epithelium during OSCC initiation. In particular, VEGFA was highly expressed in the upper layer of the epithelium at N and DN regions, but displayed no layer constraints from the DN to T region (Fig. 6b). This was in accordance with frequent communications between epithelial cells and stroma cells from the DN to T stage. To explore the higher expression of VEGFA in N and DN stage with ST feature plots and discover cellular interactions inferred by scRNA-seq, the epithelium of N and DN regions was separated into 2 parts under the guidance of pathology experts based on hyperkeratosis and dysplasia, the two identical characteristics of OLK separately initiating in the upper and lower layers of epithelium^38^ (Fig. 6c). After analysis of interactions between these two segments, we noticed that VEGFA signaling exhibited significant strength and enhanced expression in the upper layer compared to the lower layer of the N and DN region (Fig. 6d, e). This finding implied that the upper layer delivered VEGFA signaling to the proliferative cells in the lower layer, likely improving the proliferation of N and DN regions (Supplementary Fig. S9e). All above results indicated that VEGFA signaling may participate in modulating epithelial interactions by spatial-switching expression identities during OSCC initiation. Outside of epithelial VEGFA signaling, we observed CD68^+^ cells and the occurrence of CD68^+^ VEGFA^+^ signals in upper layer of N and DN regions (Fig. 6f, g). In addition to being involved in VEGFA signaling, the contributions of spatial-switching distributions of monocytic markers require further exploration.

Overall, the transcriptome and staining results demonstrated that VEGFA and TGFβ1 signalings, two hallmarks of full-blown cancer, have essential roles in OSCC initiation, with a spatial-switching regulation of VEGFA surrounding the DN stages.

### Clinical relevance of the identified immune subclusters in OSCC initiation

**Fig. 7:**
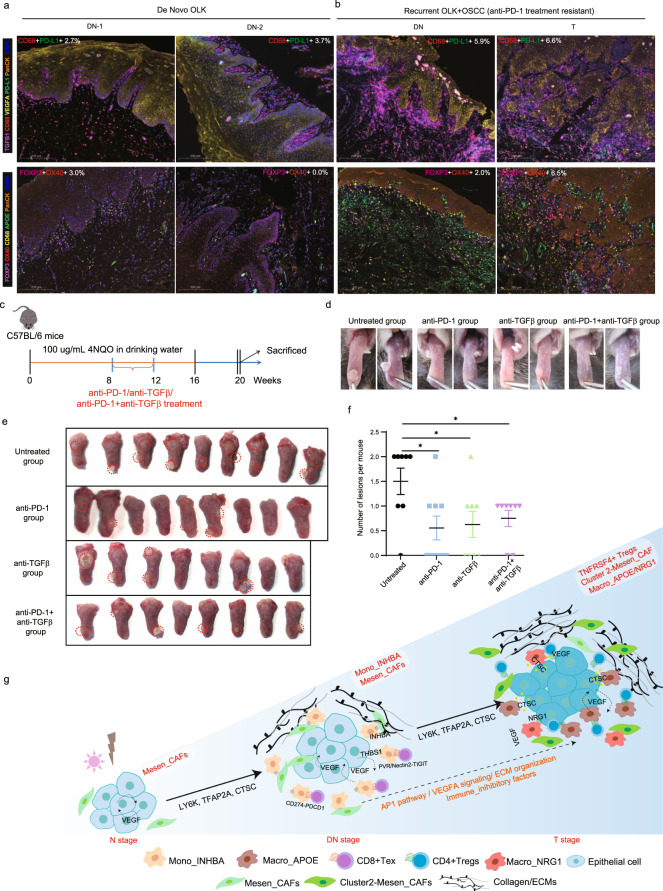
mIHC staining showing clinical relevance and cellular crosstalk landscape for OSCC initiation. **a**, **b** mIHC staining of two groups of selected markers (Group 1: PD-L1, TGFβ1, VEGFA, CD68, and PanCK; Group 2: APOE, CD68, FOXP3, OX40, and PanCK) in the samples of de novo OLK (**a**) and recurrent OSCC with OLK (**b**). Those recurrent OSCC samples with recurrent OLK were taken from patients who had OLK-derived OSCC before. The patients were under a clinical trial focusing on anti-PD-1 antibody treatment. Scale bars: 200 μm. Statistical quantification is shown in each image. **c** Flowchart showing the induction of OSCC by 4NQO in C57BL/6 mice and intraperitoneal injection of anti-PD-1/anti-TGFβ/anti-PD-1+ anti-TGFβ antibody in each treatment group. **d** Representative intraoral lesions on the tongues of mice in each group. **e** Macroscopic lesions on the tongues of each group. The dotted circle indicates cauliflower-like lesions. **f** Statistical results for quantification of the macroscopic cauliflower-like lesions in each treated and untreated group. A two-tailed Student’s *t-*test for the *P* values. ^*^P < 0.05. **g** Potential maps on malignant transformation of epithelial cells and dynamic crosstalk between epithelial cells and the TMEs during OSCC initiation.

Above discoveries on dissecting the cellular compositions and spatial organization of the OSCC initiation process will be a valuable resource to inform potential clinical strategies for cancer prevention. Clinically, moderate to severe dysplasia is correlated with recurrent OLKs, whereas mild dysplasia is often associated with de novo OLK. Resection of OLK lesions accompanied by moderate to severe dysplasia in clinics was reported to reduce relapse rate or increase the disease-free time of OLK patients^39,40^. Our data support the rationale of resecting the lesions as they locally depleted interactions with the immune-inhibitory and proliferative ecosystem. Moreover, mIHC staining confirmed different expression levels of CD68^+^ &APOE^+^ macrophages, FOXP3^+^ &TNFRSF4^+^ Tregs, VEGFA, and TGFβ signaling between the tumor microenvironment of the de novo mild OLK and the OLK with moderate to severe dysplasia in clinical study (Fig. 7a, b). The de novo OLK possessed mild TGFβ signaling and limited lymphocyte infiltration, especially immune-inhibitory myeloid cells (Fig. 7a). Instead, two biopsies with adjacent moderate to severe dysplasia showed infiltrated expression of the immune-inhibitory signals that we identified above, including CD68^+^ VEGFA^+^ monocytic cells, TNFRSF4^+^ Tregs as well as VEGF and TGFβ1 signaling (Fig. 7b). These two biopsies were taken from two poorly responded patients who were involved in a clinical study aiming to evaluate therapeutic effect of anti-PD-1 antibodies on recurrent OSCC patients with a medical history of OLK.

We next established a 4-nitroquinoline-1 oxide (4NQO)-induced carcinogenesis model in immunocompetent mice to explore the in vivo preventative functions of these identified cell subclusters. Due to the enrichment of Mono_INHBA and its frequent communications with CD8^+^Tex cells in precancerous lesions, we conducted an intervention targeting their functions with anti-TGFβ and anti-PD-1 antibodies. Groupings and induction methods are shown in a flowchart (Fig. 7c). Briefly, C57BL/6 mice were given drinking water containing 100 μg/mL 4NQO once per week for 16 consecutive weeks as previously reported^41,42^. Anti-TGFβ and anti-PD-1 antibodies were intraperitoneally injected twice a week from week 9 to week 12 (see “Materials and methods”), concurrent with precancerous lesions with mild to moderate dysplasia observation^43^ (data not shown). At week 20, we observed that mice either displayed macroscopic cauliflower-like lesions or relatively rough surface containing no obvious lesions on the tongue (Fig. 7d). After quantitation of the cauliflower-like lesions in each group, we found that all the treatment group (anti-PD-1/anti-TGFβ/anti-PD-1+anti-TGFβ group) significantly exhibited less severe lesions in comparison with the untreated group, indicating an intervention effect of these antibodies on OSCC initiation (Fig. 7e, f).

Together, our data suggested the effectiveness of an intervention therapy of anti-PD-1 antibody and inhibitors of these immune-inhibitory elements on OSCC initiation.

## Discussion

In this study, we generated data and built a single-cell and spatial transcriptome atlas of OSCC initiation. Our comprehensive analysis revealed the complex landscape of cell subclusters and spatial-switching signalings resided in the DN stage and alongside OSCC initiation (Fig. 7g and Supplementary Fig. S9f). We found that a group of initiation-associated genes were gradually enriched in epithelial cells from the N to T stage, with a promotion in epithelial proliferation and recruitment of immune-inhibitory Macro_APOE/NRG1 subclusters. Moreover, spatial-switching of VEGF signaling was observed from the upper layer of epithelium in N and DN stages to basal layers and tumor regions of epithelium in DN and T stages, with an increasing trend in communications with the surrounding ecosystem. In addition, we observed that OLK, a precancerous lesion, represented a specific stage with high enrichment of distinct monocyte subtype-Mono_INHBA. Mono_INHBA frequently interacted with CD8^+^Tex, CAFs, and epithelial cells at the DN stage, all of which were essential in promoting epithelial cell proliferation and reshaping the ECMs and immune activities favoring OSCC initiation. In addition, we identified that in the transition from DN to T stage, more functional or mature cell subclusters were involved, including cluster 2-Mesen_CAF with a progenitor phenotype, Macro_APOE/NRG1, and TNFRSF4^+^ Treg with immune tolerance activities. Together, our results indicated that future efforts could be conducted on inhibition of further enrichment of immune tolerance and epithelial cell carcinogenesis at the precancerous stage to prevent the occurrence of OSCC^41^.

A study by Hu et al. was most recently published related to single-cell RNA sequencing of 11 OSCC, 3 OLK, and 8 normal biopsies^42^. In this study, tumor samples were sampled at T2–T3 stages, while OLKs ranged from mild to severe dysplasia. The authors found a subtype of myofibroblasts that participated in the malignant transformation process. Interestingly, our data also indicated that Mesen_CAF, a subtype of fibroblasts, was involved in immune-inhibitory signaling during OSCC initiation. As the biopsies in ref. ^42^ were not from continuously progressing stages nor taken as the pairwise biopsies, it would be difficult to uncover the characteristics of DN stage or to predict initiation-associated biological processes without a very large sample size. In contrast, one strength of our study was sampling of ST and scRNA-seq biopsies from individuals simultaneously containing a normal region, moderate to severe OLK, and early OSCC at the T1 stage. It is relatively easy to uncover common variability trends in pairwise biopsies with similar medical history, which may be occluded in random sampling with high heterogeneity. The advantages of pairwise samples have been demonstrated in previous studies on exploring mutations of clonal evolution during cancer initiation process with whole exome sequencing data^43^. Notably, our study focused on deciphering the specific roles of precancerous lesions and spatial-switching elements underlying OSCC initiation, which is quite different from ref. ^42^.

Differentiated keratinocytes in the upper layer of the epithelium have often been considered as senescent cells undergoing apoptosis in clinics^44^. However, our findings reinforced their roles in enhanced secretion of VEGF signals from N to DN, likely functioning in promotion of the proliferation of lower layer basal cells. For the DN to T stage, VEGF was highly expressed in cells of basal layers and tumor regions, mainly participating in communications between endothelial, CAFs, and myeloid cells. The DN stage thus represented a stage in which epithelial cells started to interact with their surroundings. Resection at this stage may effectively block VEGF signaling and other interactions between epithelial cells and the stroma, potentially preventing its progression.

Currently, there is a lack of biomarkers or preventative strategies for OSCC intervention. Indeed, several clinical trials have been conducted aiming to intercept OSCC for patients at OLK stages, including anti-PD-1/PD-L1 therapies and inhibition of mTOR signaling^45,46^. There is an ongoing phase IIa clinical trial exploring the potential of metformin to target PI3K/mTOR signaling in oral premalignant lesions^47^. For anti-PD-1 therapies in OSCC prevention, we should mention that in September 2022, the Hanna team published their latest results on the therapeutic effect of nivolumab (an anti-PD-1 antibody) in high-risk OLK, with a favorable overall cancer-free-survival of 37%^48^. While effort is required for further assessment of the safety and effectiveness of these therapies in OSCC prevention, these studies paved the way for OSCC intervention at an early stage. In addition to mTOR inhibition and anti-PD-1/PD-L1 therapies, our results demonstrated that the involvement of immune-inhibitory myeloid cell Mono_INHBA (INHBA, TGFβ family), as well as initiation-associated VEGF, FRA, or TGFβ signaling in OSCC initiation, which may warrant clinical studies on early prevention of OSCC. In this study, we revealed the immune-inhibitory and initiation-promoting activities of the DN stage underlying OSCC initiation. Accordingly, our in vivo results proved that anti-PD-1 or inhibition of TGFβ at the DN stage was responsible for early interception of OSCC initiation.

In summary, our findings provided critical information and potential regulators for the study of unique cellular components and spatial patterns of precancerous lesions during the cancer initiation process, which paves the way for exploring cancer initiation and intervention methods in other solid cancers. The distinct and dynamic immune-inhibition elements that were newly found in this study may also be involved in the early initiation stages of other solid cancers. More attention is required to focus on immune-inhibitory myeloid, fibroblast, exhausted, and regulatory T-cell subclusters due to their reported protumor roles^49,50^. For early intervention in solid cancers at the precancerous lesion stage, these newly discovered protumor elements or cell subclusters, as well as the cancer hallmarks and clinically widely used immunotherapies will all be essential for the development of effective therapeutic strategies in cancer intervention.

## Materials and methods

### ScRNA-seq sample preparation

For scRNA-seq, fresh biopsies from individuals were firstly acquired during the surgery based on the initial clinical diagnosis and medical history of oral leukoplakia. They were immediately stored in DMEM (Corning) at 4 °C for about 30 min before the pathology characterization. After immediate H&E staining of frozen samples during the surgery, we collected samples from individuals which simultaneously contained tumor region, adjacent precancerous lesion and normal region, and each region was separated under the guidance of pathology experts at Shanghai Ninth People’s Hospital, Shanghai JiaoTong University School of Medicine. The separate regions were then digested for single-cell suspension preparation. Three indexes were included for evaluating whether the biopsies were suitable and qualified for scRNA-seq analysis, including (1) medical history of corresponding patients; (2) immediate H&E staining results of frozen samples which were very close to our biopsies; (3) the numbers of viable single cells that we gained after dissociation. Altogether, we acquired five groups of qualified scRNA-seq data for further analyses.

### ST sample preparation

For ST analyses, fresh samples containing N, DN, and T regions (based on clinical features) were delivered in Tissue Storage Solution (Miltenyi) within 1–2 h after surgery. As the capture areas of a ST slides were 6.5 × 6.5 mm, we typically chose 8 × 8 mm biopsies with the most probable area which simultaneously containing N, DN, and T regions. After removing waters on the surface of biopsies, we embedded it in optimal cutting temperature (OCT) compound (SAKURA) in a particular direction with markers at the edge of OCT frame, which helped us later getting the ideal sections. Biopsies in the OCT were quick-frozen on dry ice and stored at –80 °C before cryosection.

On the day for sample preparation, the embedded biopsies were placed in a cryostat (Leica, CM1950) for cryosection. Each tissue section was 10-μm thick. To acquire the appropriate areas for Visium Spatial slides, we chose the section under the construction of the pathology expert with quick H&E staining of series of sections. We sized the sections to match the frames of capture area on Visium Spatial slides (10× Genomics) with the aim of obtaining N, DN, and T stages in one sized section. In total, 8 sections from separate individuals were chosen based on the above procedure, whereas five sections finally met the criteria based on later H&E staining results. Among the remaining three sections, one merely presented normal and mild dysplasia and another two exhibited only T area (Supplementary Fig. S1a), possibly because the immediate analyses of H&E staining results for a 10-μm cryosection during the ST sample preparation could not reach 100% accuracy for histological analyses.

### Preparation of scRNA-seq and ST libraries and further transcriptomic analyses

Single-cell transcriptome sequencing was performed using the droplet-based 10× Genomics platform. The preparation of scRNA-seq and ST libraries and analysis of these transcriptomic data are subsequently described in Supplementary Methods.

### Analyses of The Cancer Genome Atlas (TCGA) database

Transcriptome data from TCGA_HNSCC datasets were retrieved from UCSC XENA (https://xena.ucsc.edu/). Most of the analyses were done on the GEPIA2 website (gepia2.cancer-pku.cn/)^51^. Signature genes of Mesen_CAF and TNFRSF4 + Treg cells are listed in Supplementary Table S7.

### Generation and maintenance of OSCC and OLK organoids

Human OSCC and OLK tissues were biopsied during surgical intervention. Tissues were processed as below: After washing with PBS containing 2% penicillin and streptomycin, tissues were mechanically minced into small pieces in DMEM (Corning). After centrifuging, cell pellets were re-suspended in Gentle MACs Tubes with DMEM containing 50 μg/mL Liberase™ DH (Roche), and 1 mg/mL DnaseI (Roche). After performing “human_Tumor_02” program in Gentle MACS Dissociator (Miltenyi), the tubes were then settled in a rotor at 37 °C for digestion about 30 min. Then, the suspension was strained over a 100-μm filter and centrifuged at 1000 rpm. At the same time, the remaining tissue explants with incomplete digestion were also collected. After counting cell numbers, the resulting pellets were re-suspended in ice-cold 70% matrigel at a density of 2 × 10^4^–5 × 10^4^/50 μL/well. The remaining tissue explants were also seeded.

Cultures were maintained in reported SCC culture medium at 37 °C in 5% CO_2_ and monitored daily for organoid generation. The culture medium in each well was replaced with fresh medium every 3–5 days. Organoids were passaged every 7 to 14 days at a 1:3 ratio.

### THBS1 treatment, EdU, and Ki67 quantification

For THBS1 treatment, the OLK organoids were first digested with TrypLE (Gibco). For the experiment group, cells were seeded into a 24-well plate at 2 × 10^4^ cells/well and cultured in organoid medium with 1 µg/mL THBS1 (MCE). After 10 days culture, 10 µM EdU was added to the culture system and incubated for 3 h. Cells were then dealt with the standard protocols following EdU Cell Proliferation Kit (BeyoClick) and IF staining of Ki67 with 488-conjugated florescence following the organoids IF staining protocol. Images were obtained with Zeiss 800 confocal microscope at a ×20 magnification and quantitated with Image J (National Institutes of Health, USA).

### mIHC analysis

mIHC staining was performed with the tyramide signal amplification (TSA) 6-color IHC kit (D110061-50T, WiSee Bio, China) according to the manufacturer’s instructions. Briefly, sections (3-μm thickness) obtained from paraffin-embedded samples were dewaxed, rehydrated, and subjected to 100 °C for antigen retrieval. Then, the sections were incubated with blocking antibody at room temperature for 10 min, treated with the first primary antibody for 30 min, horseradish peroxidase-conjugated (HRP) secondary antibody for 10 min, and a tyramide signal amplification for 10 min. After washing in TBST buffer, the slides went through citrate buffer antigen retrieval using a microwave set at 20% of maximum power for 15 min. The same process was repeated for the following five primary antibodies. Each slide was then treated with two drops of DAPI and manually coverslipped. Slides pictures were taken with Pannoramic MIDI tissue imaging system (3D HISTECH). Antibodies and reagents are listed in Supplementary Table S8. The distances between the interested cells were measured and quantitated with Halo Proximity Histoprogram.

### Histology and immunohistochemistry

For immunofluorescent staining of organoids, after being fixed with 4% paraformaldehyde (PFA) for 1 h, permeabilization and blocking in 3% BSA supplemented with 0.25% Triton X-100 (Sigma) for 15 min, organoids were incubated overnight with primary antibodies (listed in Supplementary Table S8) at 4 °C, washed 3 times with PBS and then incubated with fluorescence-conjugated secondary antibodies for 2 h at room temperature in the dark. Finally, the nuclei were stained with DAPI for 5 min. Images were captured using Zeiss 800 confocal microscopes (Zeiss).

For the hematoxylin and eosin (H&E) and IHC staining, tissues were fixed overnight in 4% PFA, dehydrated, cleared and embedded in paraffin as usual. In all, 3-μm-slides were sectioned and then deparaffinized, rehydrated. For IHC, citrate sodium solution (PH6) was used for antigen retrieval. After blocking endogenous peroxidase activity with 3% hydrogen peroxide for 30 min, and incubation in 1% BSA supplemented with 0.3% Triton for 1 h, primary antibodies (listed in Supplementary Table S8) were then applied at the appropriate dilutions and incubated overnight at 4 °C. The biotinylated secondary antibodies (Vector Laboratories) and the chromogen biphenyl-3,3′,4,4′-tetrayltetraammonium tetrachloride (Dako) were then added according to the manufacturer’s protocol. Fluorescence images were observed using an Zeiss BX51 microscope.

The fluorescent intensity of LY6K was analyzed in OSCC and OLK organoids, which were photographed with the same parameter setting. We used Image J software for quantification of the florescent intensity by an auto-black background setting and scoring the mean intensity of each picture. We altogether blindly analyzed 6–7 pictures from two different organoids in each group.

### Real-time PCR

TRIzol reagent (Invitrogen) was used to isolate total RNA from OLK and SCC organoids. After quantification using a SpectraMax QuickDrop™ (Molecular Devices), a total of 0.8 μg RNA was reverse transcribed into cDNA using M-MLV reverse transcriptase (Takara). Quantitative PCR was then performed using SYBR Premix Ex Taq on a Roche LightCycler 96 Real-time PCR system. *GAPDH* was used as an endogenous control for all the genes. The primer sequences are listed in Supplementary Table S8.

### Gene knockdown by siRNAs

Three specific siRNAs for each target gene (Supplementary Table S8) were designed and synthesized by Genomeditech. For siRNA transfection of cells in the 24-well format, 100 nM (final concentration) siRNA was transfected in serum-free Opti-MEM (Gibco) with 2 μL of Lipofectamine 3000 (Invitrogen) for each well. After 8–10 h of transfection, we changed medium and cultured for another 24 h in complete culture media to characterize RNA expression level.

### 4NQO-induced oral tumorigenesis

6-week-old male C57BL/6 mice were purchased from Shanghai Jihui Laboratory Animal Care Co., Ltd. and were chosen for induction of OSCC with 4NQO. All mice were given sterile water containing 100 μg/mL 4NQO (Sigma-Aldrich) for 16 consecutive weeks. From week 16 to week 20, the 4NQO was replaced with sterile water. Mice received 200 mg anti-PD-1 antibody (InVivoMAb, BioXCell, anti-mouse-PD-1 antibody, BE0146, clone RMP1-14) and 300 mg anti-TGFβ antibody (InVivoMAb, Selleck, anti-human/mouse-TGFβ antibody, A2113, clone 1D11.16.8) per injection intraperitoneally. All the antibody or antibody combinations were given twice a week and altogether for 4 weeks from week 9 to week 12 of induction. The experiment lasted until the end of week 20 and was performed in a specific pathogen-free environment. Mice were sacrificed at week 20, and the tongue lesions were collected for histopathological analysis, while the spleens were harvested for further flow cytometry.

### Statistics and reproducibility

All analyses of scRNA-seq and ST were performed using R package (V.4.0.3) with statistical significance set at *P* < 0.05 adjusted for multiple testing. When assessing differently expressed genes with Seurat, a nonparametric Wilcoxon rank-sum test was applied. Kaplan–Meier curves with log-rank statistics were used to compare overall survival. Pearson correlation analysis for TCGA bulk RNA-seq and one-way ANOVA test for differential analyses were performed using GEPIA2. To determine the significance of percentage changes or gene-expression variations between two groups, an unpaired or paired two-sided *t* test was used with Microsoft Excel or GraphPad Prism 8 software. For other statistical significance values and sample sizes in graphs, see the figure legends and “Materials and methods” for details. Most of the experiments were repeated three times independently, with similar results, and detailed information has been provided in each figure legend.

### Study approval

All experimental procedures related to sample collection and operation were approved by the Institutional Review Board of Shanghai Ninth People’s Hospital, Shanghai Jiao Tong University School of Medicine, with Research Ethics Board approval in accordance with the Declaration of Helsinki. Written informed consent was obtained from all study participants. All animal experiments were approved and performed in accordance with the guidelines of the Institutional Review Board and Animal Care and Use Committee of Shanghai Ninth People’s Hospital, Shanghai Jiao Tong University School of Medicine.

## Supplementary information

Supplementary Figures and methods

Supplementary Table S1

Supplementary Table S2

Supplementary Table S3

Supplementary Table S4

Supplementary Table S5

Supplementary Table S6

Supplementary Table S7

Supplementary Table S8

## Data Availability

All data are available in the main text, figures or other supplementary materials. The additional data is also available from the corresponding author upon reasonable request. All the raw data of the scRNA-seq and ST analyses generated in this study were deposited in the Genome Sequence Archive (GSA) in the BIG Data Center (https://ngdc.cncb.ac.cn/gsa-human/), Beijing Institute of Genomics (BIG), Chinese Academy of Sciences, under accession number HRA004032 and HRA004033.
