# Supplementary material for: Single-cell and spatial dissection of precancerous lesions underlying the initiation process of oral squamous cell carcinoma
Source: Cell Discov. 2023 Mar 13;9:28. doi: 10.1038/s41421-023-00532-4 (PMC10011538; doi:10.1038/s41421-023-00532-4)
Supplement: Supplementary file 1 — Supplementary Figures and methods [file 41421_2023_532_MOESM1_ESM.pdf]

Supplementary Materials

For

Single-cell and spatial dissection of precancerous lesions underlying the initiation process of oral squamous cell carcinoma

Lulu Sun[1,2#], Xindan Kang[1,2#], Chong Wang[1,2], Rui Wang[1,2], Guizhu Yang[1,2], Wen Jiang[2,3], Qi Wu[1,2], Yujue Wang[1,2], Yaping Wu[1,2], Jiamin Gao[1,2], Lan Chen[1,2], Jie Zhang[1,2], Zhen Tian[2,4], Guopei Zhu[2,3], Shuyang Sun[1,2]*

Corresponding author. Email: sunshuyang@sjtu.edu.cn (S.S.Y.)

The PDF file includes:

Supplementary Methods and Supplementary Figures. S1-S9

Supplementary Table legends for Supplementary Tables. S1-S8

Other Supplementary Material for this manuscript includes the following:

Supplementary Tables. S1 to S8

Supplementary Methods

Preparation of single-cell suspensions

Briefly, the biopsies were first washed with phosphate-buffered saline (PBS), mechanically dissociated with a scalpel into small pieces (approximately 1mm^3) on ice, then enzymatically dissociated with human-tumor dissociation kit (Miltenyi) for 30-45 min (30 min for tumor area, and 45 min for normal and dysplasia area) in the shaker at 37℃. After dissociation, cells were neutralized with PBS, sieved through a 70 µm cell strainer (BD falcon), and centrifuged at 400g for 5 min. Then, the cell pellet was re-suspended in 1 mL of ice cold red blood cell lysis buffer (Miltenyi) at 4℃ for 5 min to remove red blood cells. Cells were then filtered using a 35-µm nylon mesh (BD falcon) after washing with PBS. Next, the single cells were stained with AO/PI for viability assessment with the Countstar Fluorescence Cell Analyzer.

Single-cell Sequencing

The scRNA-seq libraries were constructed with the 10x Genomics Chromium Controller Instrument and Chromium Single Cell 3' V3.1 Reagent Kits (10x Genomics, Pleasanton, CA) according to the manufacturer's instructions. Briefly, cells were loaded with a concentration about 1000 cells/µL into the microfluidic chips for generating single-cell Gel bead-in emulsions (GEM). The barcoded-cDNA in the GEM was then purified and amplified with the RT step. Next, the amplified barcoded cDNA was fragmented, A-tailed, ligated with adaptors and index PCR amplified for generating libraries. Followed by the standard process for library construction, the final libraries were then quantified with the Qubit High Sensitivity DNA assay (Thermo Fisher Scientific). The size distribution was determined with a High Sensitivity DNA

chip on a Bioanalyzer 2200 (Agilent). Sequencing of the libraries were done on an Illumina NovaSeq 6000 sequencer (Illumina, San Diego, CA) with a 150 bp paired-end run.

Staining and imaging on Visium Spatial slides

The section slides of all the biopsies were incubated 1 min at 37°C followed by fixation in methanol at -20°C for 30 min. Upon H&E staining, slides were incubated in hematoxylin for 7 min, Bluing Buffer for 2 min and eosin for 1 min. Afterwards, slides were washed with DNase and RNase free water before imaging with the microscope (Pannoramic MIDI, 3DHISTECH).

Tissue permeabilization and spatial transcriptomic sequencing

In order to acquire the optimal permeabilization time, we have done pre-permeabilization on 4 sections among these 8 sections (2 sections for one slide). Tissue Optimization Slides & Reagent Kits (10x Genomics) were used for pre-permeabilization. Sections were permeabilized in Permeabilization Enzyme for 3 min, 6 min, 12 min, 18 min, 24 min and 30 min before the Fluorescent RT Master Mix was added. Then, the sections were incubated in Tissue Removal Mix for 60 min at 56°C for tissue removal. The best permeabilization time was selected based on the fluorescent strength. For formal experiments, permeabilization and spatial transcriptomic sequencing were performed using Visium Spatial Gene Expression Slides & Reagent Kits. The stained slides were incubated in RT Master Mix for 45 min at 53 °C for reserve transcription after permeabilization for 24 min. Followed by the standard process for library construction, the final libraries were then quantified and sequenced with similar process in scRNA-seq.

ScRNA-seq and ST Statistical Analysis

ScRNA-seq and ST data analysis was performed by NovelBio Co., Ltd. with NovelBrain Cloud Analysis Platform (www.novelbrain.com). The sequencing results were filtered with FASTP for filtering the adaptor sequence and removing low-quality reads [1]. Then the feature-barcode matrices were obtained by aligning reads to the human genome (GRCh38) with CellRanger v3.1.0 for scRNA-seq and SpaceRanger v1.1.0 for ST analyses.

ScRNA-seq Data Visualization

Based on the mapped barcoded reads per cell in each sample, we applied the down sample analysis among samples to achieve the aggregated matrix. Criteria for cell quality filtering: over 200 genes expressed in a cell + mitochondria UMI rate below 20%. Mitochondria genes were then removed in the expression table. Then, single-cell expression table was acquired according to the UMI counts of each sample and percent of mitochondria rate. To obtain the scaled data, the following cell normalization and regression were then done with Seurat package (version: 3.1.4, https://satijalab.org/seurat/) based on the expression table. Top 2000 high variable genes were then used for PCA construction. TSNE and UMAP were then constructed with top 10 principal components of PCAs. The unsupervised cell cluster results were done with graph-based cluster method, and the marker genes and DEGs were calculated by FindAllMarkers function with Wilcox rank sum test algorithm under following criteria:1. lnFC > 0.25; 2. P-value<0.05; 3. min.pct>0.1.

For detailed cell subtype identification, the single-cell data within major cell types were selected for re-TSNE analysis, and the following graph-based clustering and marker analysis.

ST Data Visualization

Spot normalization and regression was done with Seurat package (version: 3.2, https://satijalab.org/seurat/). PCA and tSNE protocols were similar to scRNA-seq analyses. Spatial feature expression plots were generated with the SpatialFeaturePlot function in Seurat (version 3.1.4) and the STUtility R package (version 1.0.0).

Pseudo-time analysis

Single-cell trajectories analysis was conducted with Monocle2 (http://cole-trapnell-lab.github.io/monocle-release) using DDR-Tree and default parameter. Before Monocle analysis, we select marker genes of the Seurat clustering result and raw expression counts of the cell passed filtering.

RNA velocity

RNA Velocity analysis was conducted by the scVelo (Version 0.2.3) method in the ScanPy Python package with default parameters for inferring the cell dynamics [2].

SCENIC analysis

We applied the single-cell regulatory network inference and clustering (pySCENIC, v0.9.5) workflow with the 20-thousand motifs database for RcisTarget and GRNboost to evaluate transcription factor regulation strength [3].

CNV estimation

Endothelial, fibroblast and macrophage cells were used as reference to identify somatic CNVs with the R package inferCNV (v0.8.2) [4]. We scored the extent of CNV signal in each

cell and defined it as “the mean of squares of CNV values” across the genome. Putative malignant cells were then defined with criterion: CNV signal above 0.05 and CNV correlation above 0.5. The corresponding genes in these CNV variable regions were inferred with inferCNV (1.12.0) and HMM models.

Cell communication analysis

We conducted cell communication analyses based on the CellPhoneDB [5], a public repository of ligands, receptors and their interactions, to enable a systematic prediction of cell-cell communication molecules. Membrane proteins, secreted and peripheral proteins were annotated. Significant mean and cell communication significance (*P*-value<0.05) were calculated based on the interaction and the normalized cell matrix achieved by Seurat Normalization. All the interaction pairs that we listed in the manuscript were all of significance.

Cell type scoring

To analyze the distribution of cell types in N, DN and T areas of the biopsy sections, we scored the cell type enrichment in each spot with a signature-based strategy. First, we selected several uniquely-expressed marker genes as the signature genes with FindAllMarkers function. Next, GSVA (1.32.0) was used to assign signature score estimates to individual spots [6]. Taking advantage of these scores, relative enrichment degree of different cell types could then be compared.

QuSAGE analysis (Gene Enrichment Analysis)

QuSAGE (2.16.1) analysis was performed to evaluate the activation features of a given gene set such as pathway activation [7]. QuSAGE accounts for inter-gene correlations using the

Variance Inflation Factor technique.

Gene set enrichment analysis

Gene set enrichment analysis (GSEA) using Gene Ontology (GO) annotations was performed to elucidate the biological implications of marker genes and differentially expressed genes [8]. GO annotations and gene sets information were downloaded from GSEA website (https://www.gsea-msigdb.org/gsea/msigdb/genesets.jsp) [9,10]. Fisher's exact test was applied to identify the significance and false discovery rate (FDR) was calculated to correct the *P*-values due to multiple tests. GSEA analysis in Figure 1f was performed using Metascape (http://metascape.org) [11,12] and the *P*-values were derived from a hypergeometric test and Benjamini–Hochberg correction was used to adjust the *P*-values for identifying the significantly enriched GO terms.

Correlation heatmap analysis

The correlation analysis was performed in main cell types and epithelial subclusters of diverse initiation stage using (pheatmap) with a calculation of "Pearson correlation". By doing so, cells were then distributed close by their correlations among each subclusters. We classified all these subclusters of N, DN and T stages into 4 clusters. Cluster 1, common genes among these stages; Cluster 2, higher expression in N and DN stages; Cluster 3-1 and 3-2, genes that were higher expression in DN and T stages; Cluster 4, genes with higher expression in T. The marker genes of each cluster were selected based on the co-expression of these genes between the correlation results and the heatmap results. All the markers were then confirmed with ST feature plots.

References ***(1-12)***

1 Chen, S., Zhou, Y., Chen, Y. & Gu, J. fastp: an ultra-fast all-in-one FASTQ preprocessor. *Bioinformatics* **34**, i884-i890, doi:10.1093/bioinformatics/bty560 (2018).

2 Wolf, F. A., Angerer, P. & Theis, F. J. SCANPY: large-scale single-cell gene expression data analysis. *Genome Biol* **19**, 15, doi:10.1186/s13059-017-1382-0 (2018).

3 Aibar, S. *et al.* SCENIC: single-cell regulatory network inference and clustering. *Nat Methods* **14**, 1083-1086, doi:10.1038/nmeth.4463 (2017).

4 Tickle, T., Tirosh, I., Georgescu, C., Brown, M., & Haas, B. inferCNV of the Trinity CTAT Project. Klarman Cell Observatory, Broad Institute of MIT and Harvard. https://github.com/broadinstitute/inferCNV. (2019).

5 Vento-Tormo, R. *et al.* Single-cell reconstruction of the early maternal-fetal interface in humans. *Nature* **563**, 347-353, doi:10.1038/s41586-018-0698-6 (2018).

6 Hanzelmann, S., Castelo, R. & Guinney, J. GSVA: gene set variation analysis for microarray and RNA-seq data. *Bmc Bioinformatics* **14**, 7, doi:10.1186/1471-2105-14-7 (2013).

7 Yaari, G., Bolen, C. R., Thakar, J. & Kleinstein, S. H. Quantitative set analysis for gene expression: a method to quantify gene set differential expression including gene-gene correlations. *Nucleic Acids Res* **41**, e170, doi:10.1093/nar/gkt660 (2013).

8 Ashburner, M. *et al.* Gene ontology: tool for the unification of biology. The Gene Ontology Consortium. *Nat Genet* **25**, 25-29, doi:10.1038/75556 (2000).

9 Subramanian, A. *et al.* Gene set enrichment analysis: a knowledge-based approach for interpreting genome-wide expression profiles. *Proc Natl Acad Sci U S A* **102**, 15545-15550, doi:10.1073/pnas.0506580102 (2005).

10 Liberzon, A. *et al.* The Molecular Signatures Database (MSigDB) hallmark gene set collection. *Cell Syst* **1**, 417-425, doi:10.1016/j.cels.2015.12.004 (2015).

11 Zhou, Y. *et al.* Metascape provides a biologist-oriented resource for the analysis of systems-level datasets. *Nat Commun* **10**, 1523, doi:10.1038/s41467-019-09234-6 (2019).

12 Mootha, V. K. *et al.* PGC-1alpha-responsive genes involved in oxidative

phosphorylation are coordinately downregulated in human diabetes. *Nat Genet* **34**, 267-273, doi:10.1038/ng1180 (2003).

Supplementary Figures

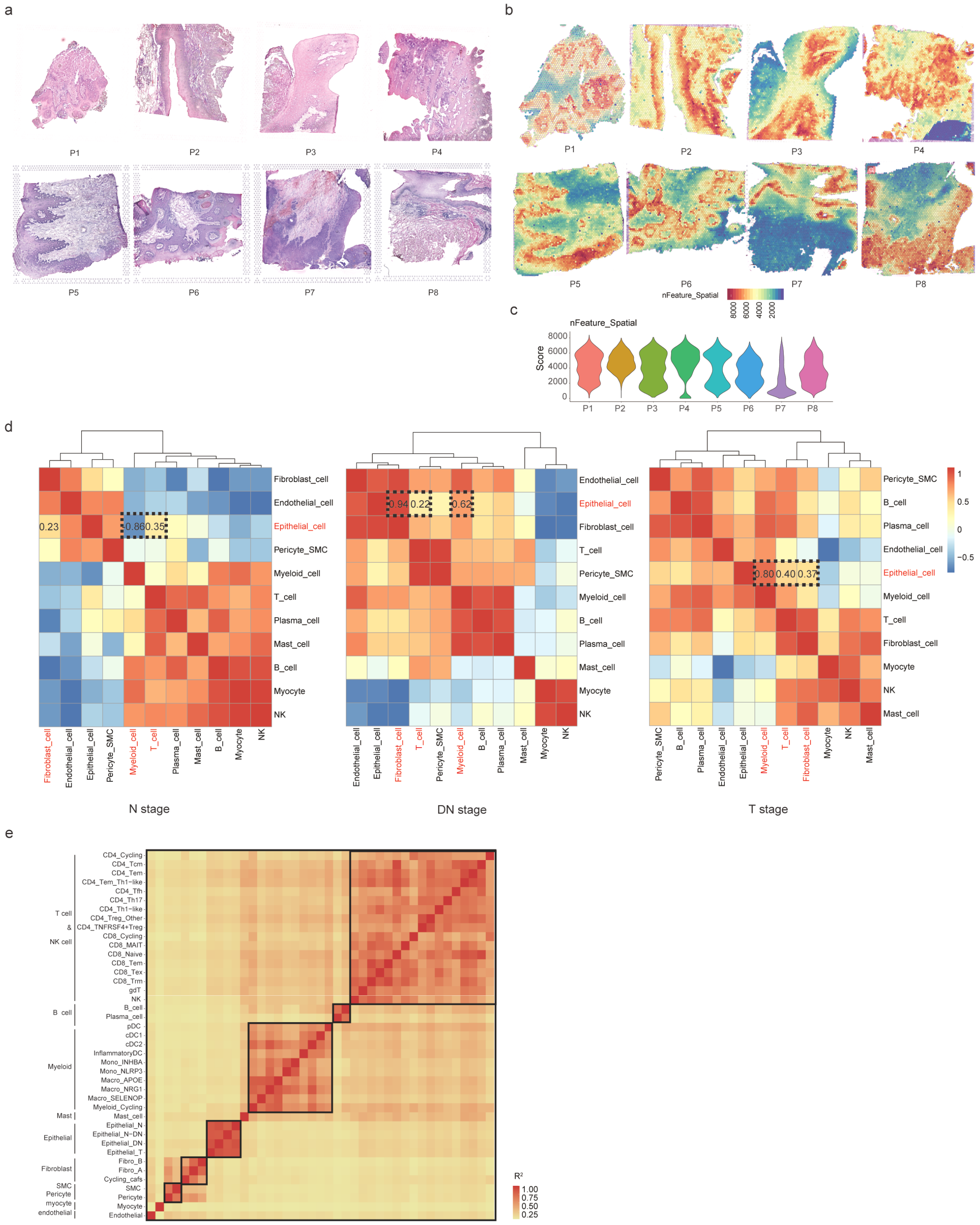

Supplementary Fig. S1

Supplementary Fig. S1. Spatial feature plots and transcriptome data analysis.

(a) Spatial feature plots of ST samples. **(b)** Spatial feature plots of the numbers of expressed transcripts (nUMIs) and genes (nGene). **(c)** Score of the number of expressed genes (n Feature_Spatial) for each

ST sample. **(d)** Correlation heat map of major cell types which were identified in scRNA-seq with ST data using Pearson correlation matrix at N regions (left); DN regions (middle) and T regions (right). The numbers in the heatmap represented correlation index. **(e)** Cluster–cluster heat map of gene-expression data for markers of identified cell types across all samples of scRNA-seq using Pearson correlation matrix. Darker colors correspond to higher correlation.

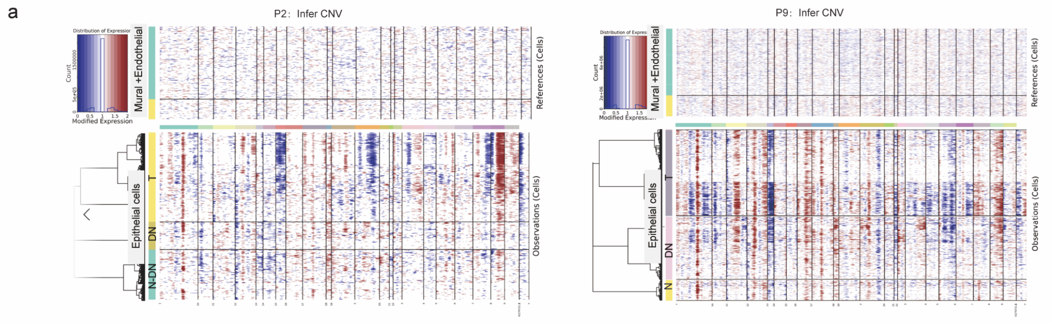

b

	P2			P9			P10		P11		P12		
	N-DN	DN	T	N	DN	T	N-DN	T	DN	T	N	DN	T
MTOR (chr 1)	—	—	—	—	—	Amp (4)	—	Amp (4)	Amp (4)	Amp (4)	Amp (4)	Amp (4)	—
EGFR (chr 7)	—	Amp (4)	Amp (4)	—	—	—	Amp (4)	Amp (4)	Amp (4)	Amp (4)	—	Amp (4)	Amp (4)
MYC (chr 8)	—	—	Amp (4)	—	—	—	Amp (4)	Amp (4)	Amp (4)	Amp (4)	Amp (4)	Amp (4)	Amp (4)
CCND1 (chr 11)	—	—	—	—	Amp (4)	Amp (4)	Amp (4)	Amp (4)	Amp (4)	Amp (4)	Amp (4)	Amp (4)	Amp (4)
RB1 (chr 13)	—	—	—	Del (2)	Del (2)	Del (2)	Del (2)	Del (2)	—	Del (2)	—	—	Del (2)
TOP2A (chr 17)	Amp (4)	Amp (4)	Amp (4)	—	—	—	Amp (4)	Amp (5)	—	—	Amp (4)	Amp (4)	Amp (5)
ERBB2 (chr 17)	Amp (4)	Amp (4)	Amp (4)	—	—	—	Amp (4)	Amp (5)	—	—	—	—	—
TFAP2A (chr 6)	—	—	—	—	—	—	Amp (4)	—	—	—	—	—	—
LY6K (chr 8)	—	—	Amp (5)	Amp (4)	Amp (4)	Amp (5)	Amp (4)	Amp (4)	\	\	\	\	\
FADD (chr 11)	Amp (4)	Amp (4)	—	—	Amp (4)	Amp (4)	Amp (5)	Amp (5)	\	\	Amp (4)	Amp (4)	Amp (4)
PMEPA1 (chr 20)	—	Amp (4)	—	—	—	Amp (4)	—	—	—	—	Amp (4)	Amp (4)	Amp (4)

(chr: chromosome; Amp: CNV amplification; Del: CNV deletion; '\': invariant by inferCNV;'-': Score 3 for no CNV amplification or deletion in HMM model ; '(2)': Score 2 for CNV deletetion in HMM model; '(4)(5)': Score 4 or 5 for CNV amplificationin HMM model, 5 means higher CNVamplification;)

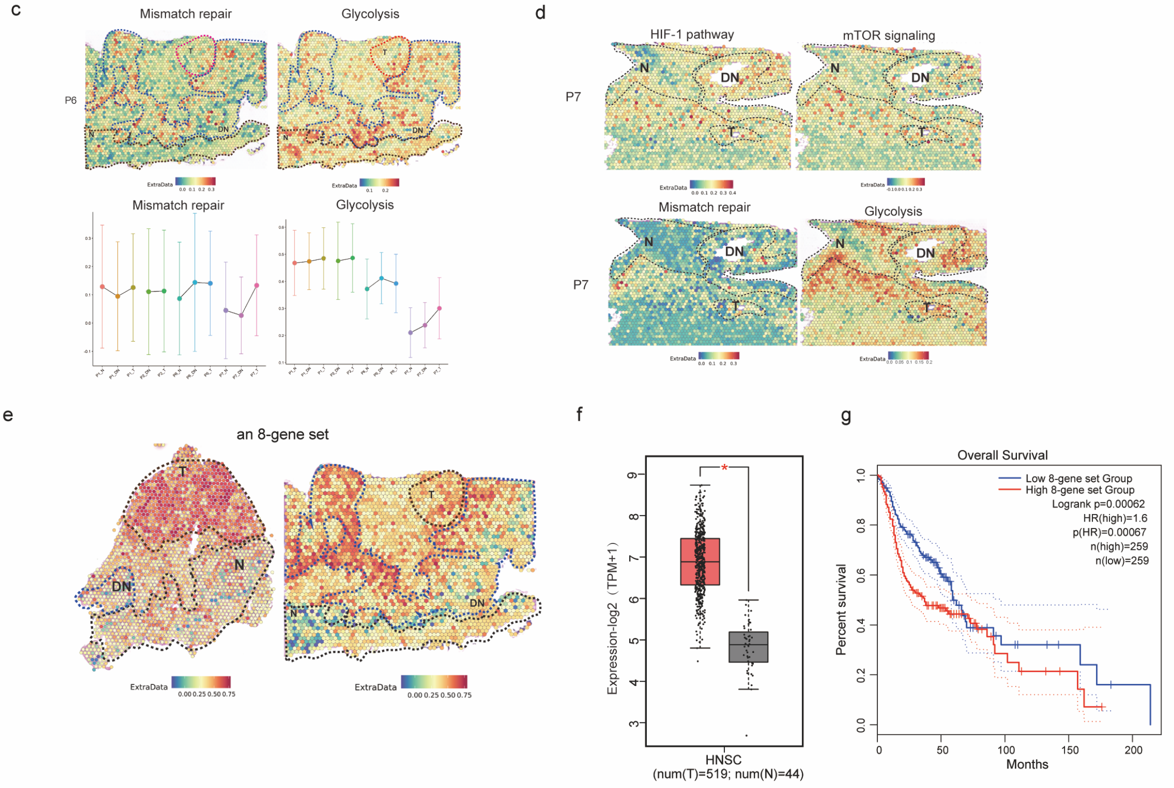

Supplementary Fig. S2

Supplementary Fig. S2. InferCNV and pathway analyses in epithelial cell subtype.

(a) Heatmap shows large-scale CNVs for individual cells (rows) from patient 2 and patient 9, which were done with "inferCNV" package. Red: amplifications; blue: deletions. Endothelial and

pericytes/SMCs as normal cells as a reference. **(b)** The table illustrated recurrent CNVs in regions with well-recognized cancer driver and tumor suppressor genes, using scRNA-seq data analyzed by the "inferCNV" package and "HMM models". **(c)** Characterization of mismatch repair and glycolysis process with ST feature plots from P6 (top). Changes of the two pathways' activities along with tumor initiation process in patients of ST (bottom). Each dot indicated the median of the pathway activity in the corresponding region. (**d**) Characterization of OSCC initiation process with ST feature plots from P7, showing cancer-related pathways. **(e)** Dot plot of selected 8 marker genes as a whole (an 8-gene set) which were previously reported to be potentially associated with OSCC initiation. **(f)** Comparison of the expression levels of the 8 initiation-associated gene sets between normal and tumor tissues with TCGA_HCC cohort data, which were done on GEPIA2 website. One-way ANOVA tests were used to analyze the significance of their differences. *P<0.05. **(g)** Kaplan–Meier survival curve for patients of TCGA_HCC cohort, grouped by higher and lower expression of the 8 initiation-associated gene sets, which were done with GEPIA2 website. Log-rank statistics were used to compare overall survival.

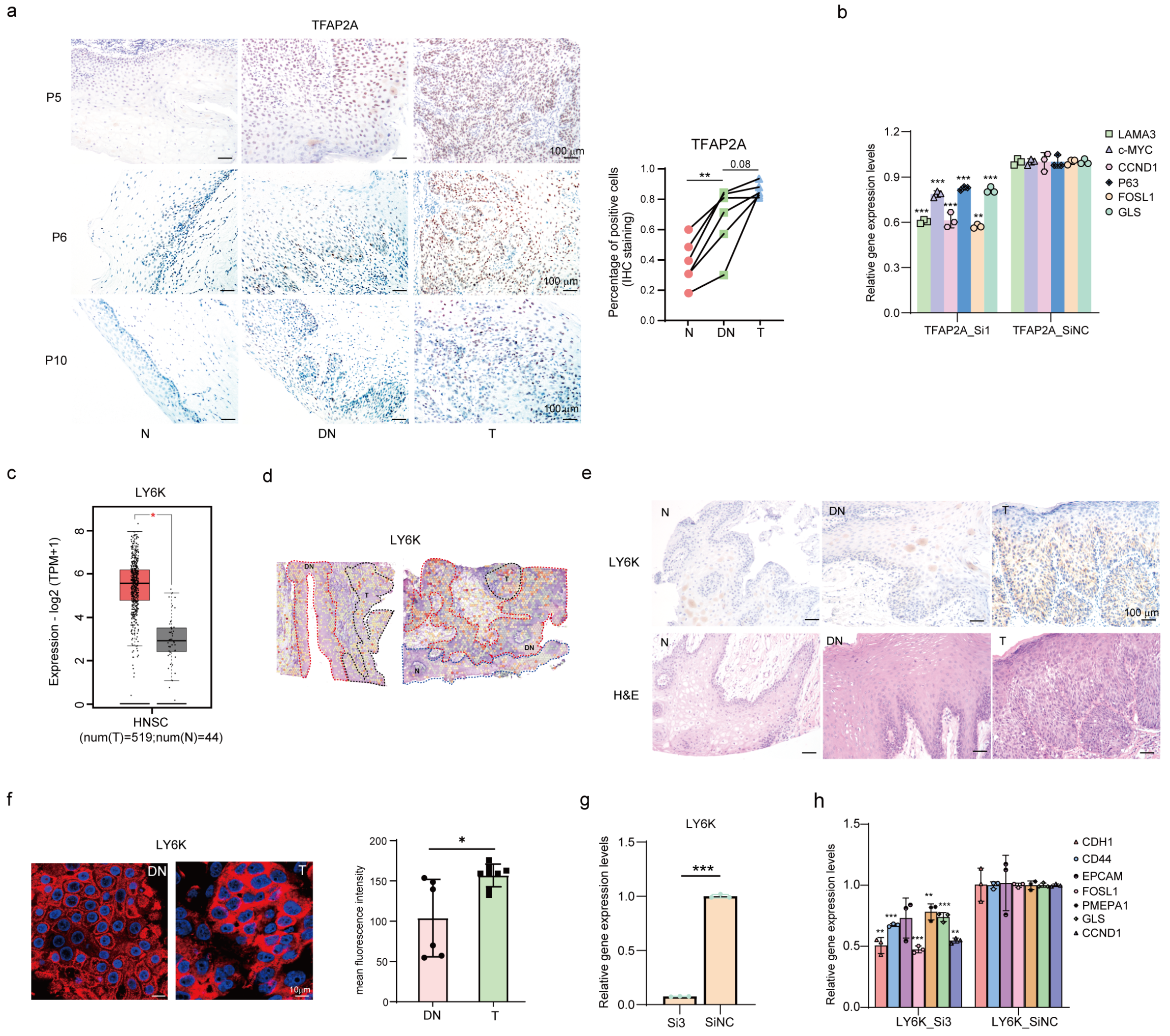

Supplementary Fig. S3

Supplementary Fig. S3. Identification and characterization of initiation-associated genes and pathways.

(a) Representative IHC staining results of TFAP2A in patients (left). Scale bar: 100 µm. Statistical analysis showing the percentage of TFAP2A positive cells in each region (right). A two-tailed paired Student's t-test for the *P*-values. ***P*< 0.01. **(b)** The expression levels of initiation-associated genes were down-regulated in siTFAP2A group. ***P*< 0.01; ****P*< 0.001. **(c)** Comparison of the expression levels of LY6K between normal and tumor tissues with TCGA_HCC cohort data, which were done on GEPIA2 website. One-way ANOVA tests were used to analyze the significance of their differences. **P*<0.05. (**d**) Spatial feature plots of LY6K in tissue sections of P2 and P6. **(e)** Representative IHC (top) staining of LY6K and the corresponding H&E result (bottom) in different initiation stages of a patient. Scale bar: 100 µm. **(f)** Representative immunofluorescent (IF) staining of LY6K in OLK (left) and OSCC organoids (right). Scale bar: 10 µm. **(g)** The gene expression levels of LY6K in siLY6K and

siNC group. Data presented as the mean ± s.d. A two-tailed unpaired Student's t-test for the *P*-values. ***P<0.001. NC, control group. **(h)** The expression levels of initiation-associated genes were down-regulated in siLY6K group. A two-tailed unpaired Student's t-test for the *P*-values. *P< 0.05; **P< 0.01; ***P< 0.001.

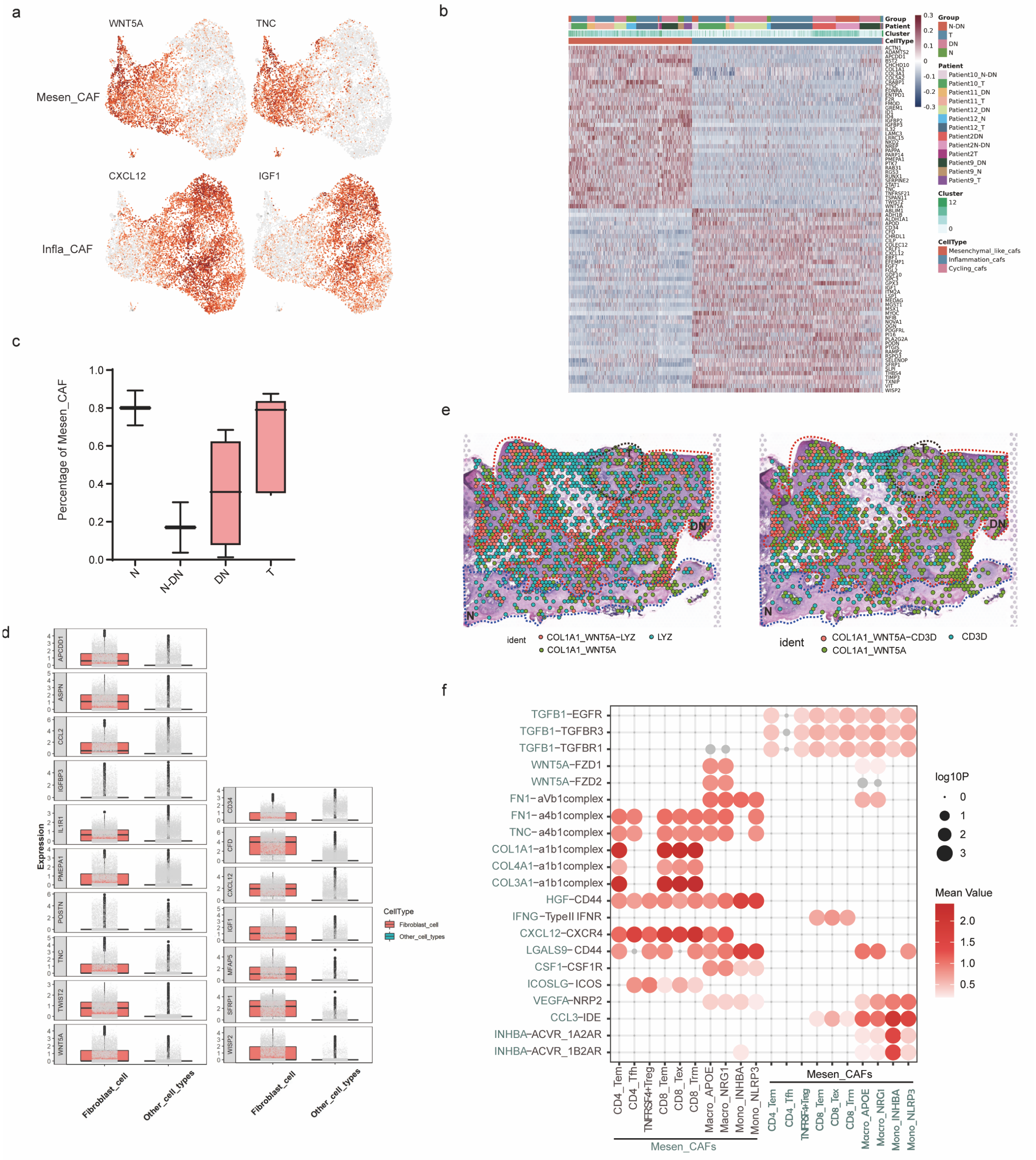

Supplementary Fig. S4

Supplementary Fig. S4. Characteristics of identified Mesen_CAFs and Infla_CAFs by scRNA-seq.

(a) UMAP feature plots showing expression distribution of Mesen_CAF marker WNT5A and Infla_CAF marker IGF1 in single fibroblast cell. **(b)** Heatmap of genes with differential expression (rows) between Mesen_CAF and Infla_CAF subclusters. **(c)** Statistics showing 2 fibroblast subpopulations in each initiation stage with scRNA-seq data. **(d)** Comparison of the expression levels

of identified fibroblast subcluster markers (Mesen_CAF marker: left; Infla_CAF marker: right) between fibroblasts and other types of cells with scRNA-seq data. Two-sided Wilcoxon rank sum tests were used to analyze the significance of their differences. ***P<0.001. **(e)** Spatial feature plots for Mesen_CAF marker (COL1A1+ & WNT5A+), myeloid cell marker (LYZ+) and T cell marker (CD3D+). Red dots indicated coexpression. **(f)** CellphoneDB analysis of inferred interactions between Mesen_CAFs and immune cell subclusters. Dotplots showing the significance (−log10 P-value) and strength (mean value) of specific interactions. Mean and significance (P-value<0.05) were calculated based on the interaction and the normalized cell matrix achieved by Seurat Normalization.

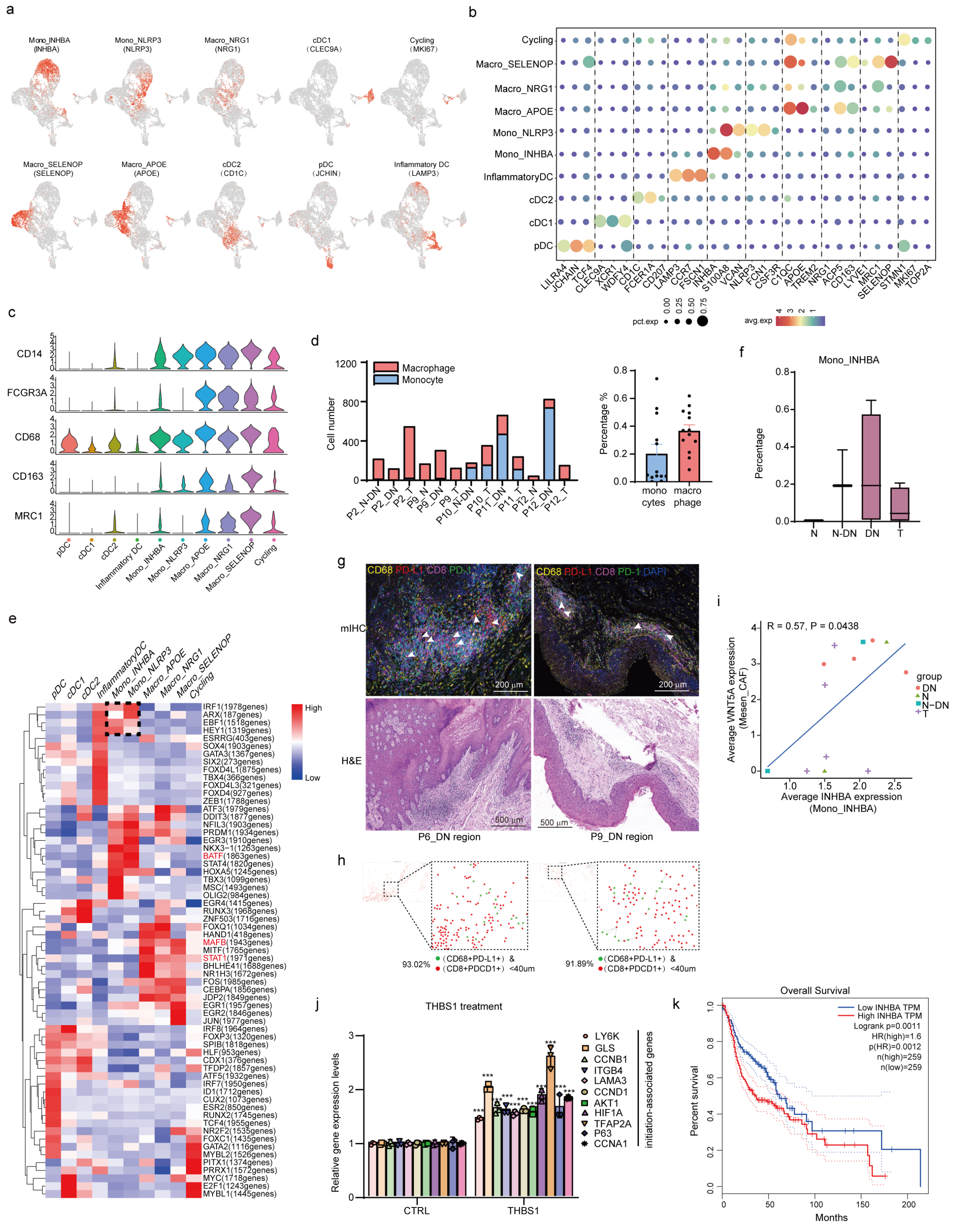

Supplementary Fig. S5

Supplementary Fig. S5. Cell proportion and functional characterization of myeloid cells subpopulations with scRNA-seq data.

(a) UMAP feature plots showing expression distribution of myeloid subcluster markers. **(b)** Bubble heatmap showing expression levels of selected signature genes in identified myeloid subclusters. Dot size indicates fraction of expressing cells, colored based on normalized expression levels. **(c)** Violin plots showing expression levels of macrophage markers across different myeloid cell types from scRNA-seq data. **(d)** Cell numbers and proportions of monocytes and macrophages in patients of OSCC initiation by scRNA-seq data. (left) presented with single patient; (right) presented as a whole. **(e)** Heatmap showing the activity of TFs in each myeloid subpopulation. The TF activity is scored with SCENIC package. **(f)** Cell proportions of Mono_INHBA in diverse initiation stages by scRNA-seq data. **(g)** mIHC staining of Mono_INHBA marker (CD68+ & PD-L1+) and CD8_Tex marker (CD8+ & PDCD1+) in the DN stages of patients, scale bars: 200 μm (top). H&E staining of DN stages of patients, scale bars: 500 μm (bottom). **(h)** Statistical analysis showing distances between Mono_INHBA and CD8_Tex cells. Note that the distances between over 90 % of Mono_INHBA and CD8_Tex cells were within 40 μm. **(i)** Pearson correlation plot showing significant positive correlation of WNT5A expression in Mesen_CAF to INHBA expression in Mono_INHBA. Cor.test() was used for conducting correlation and the statistical analyses. **(j)** Expression levels of initiation-associated genes upon THBS1 (1μg/mL) treatment in OLK organoids for 10 days. A two-tailed unpaired Student's t-test for the *P*-values. **P*< 0.05. ***P*< 0.01. ****P*< 0.001. **(k)** Kaplan–Meier survival curve for patients of TCGA_HNSCC cohort, grouped by higher and lower expression of INHBA, which were done with GEPIA2 website. Log-rank statistics were used to compare overall survival.

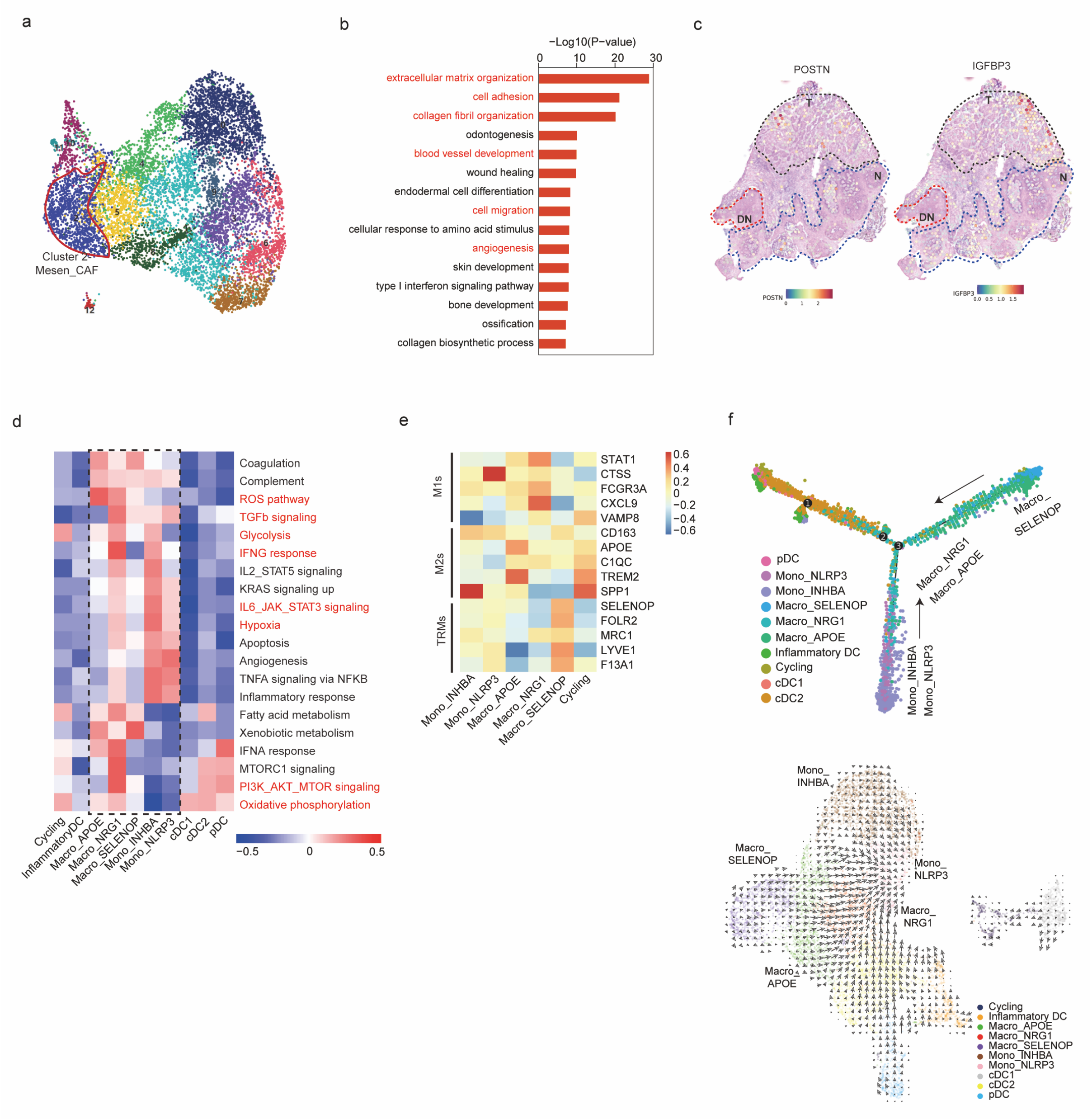

Supplementary Fig. S6

Supplementary Fig. S6. Characterization of cluster 2-Mesen_CAF and Macro_ APOE / NRG1 subclusters with scRNA-seq and ST data.

(a) UMAP representation of fibroblasts. Note that Cluster 2-Mesen_CAF were circled with red color. **(b)** Enriched GO terms for Cluster 2-Mesen_CAFs. Fisher's exact test was applied to identify the significance and FDR was used to correct the *P*-values. **(c)** Spatial feature plots of Cluster 2-Mesen_CAF markers (POSTN, IGFBP3) in tissue sections of P1. **(d)** Enriched hallmark gene sets in each myeloid subpopulation done with QuSAGE. **(e)** Heatmap showing expression levels of selected markers of M1, M2 and TRMs in each myeloid subpopulation. **(f)** Potential Trajectory of differentiation from monocyte into Tumor-associated macrophages inferred by analysis with Monocle 2 (top).

Trajectory with arrows predicting the directions of certain monocytes and macrophages properties annotated with the signatures. RNA velocities represented by arrows overlaid on the UMAP plots showing the transition potential of myeloid subclusters (bottom). Each dot for a single cell.

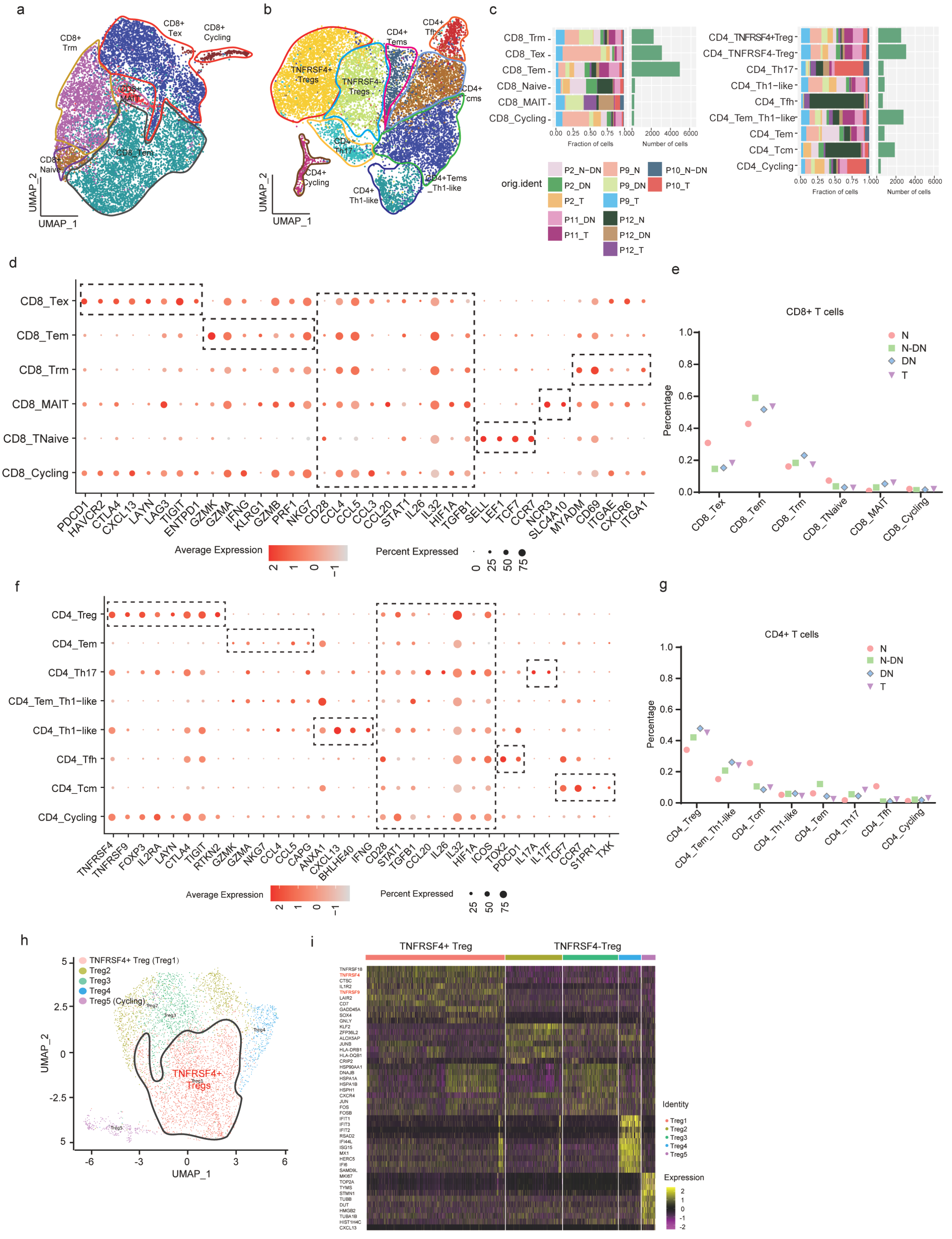

Supplementary Fig. S7

Supplementary Fig. S7. Profiling of CD8+ and CD4+ T cell subpopulations with scRNA-seq data.

(a-b) UMAP representation of single T cells colored by subclusters (left, **a**), CD8A and CD4 expression levels (right, **b**). **(c)** Bar plots of T cell subpopulations in each scRNA-seq sample (left) and the corresponding number of cells, UMIs and genes in each group (right). **(d)** Bubble heatmap showing expression levels of selected signature genes in different CD8+ T cell subpopulations. Dot size indicated fraction of expressing cells, colored based on normalized expression levels. **(e)** Cell proportions of CD8+T cells in patients of diverse initiation stages by scRNA-seq data. **(f)** Bubble heatmap showing expression levels of selected signature genes in different CD4+ T cell subpopulations. Dot size indicated fraction of expressing cells, colored based on normalized expression levels. **(g)** Cell proportions of CD4+T cells in patients of diverse initiation stages by scRNA-seq data. Heatmap showing the activity of TFs in each T cell subpopulations. The TF activity was scored using SCENIC analyses. **(h)** UMAP representation of single CD4+Treg cells colored by identified cell types. **(i)** Heatmap showing expression of marker genes in different Treg subpopulations.

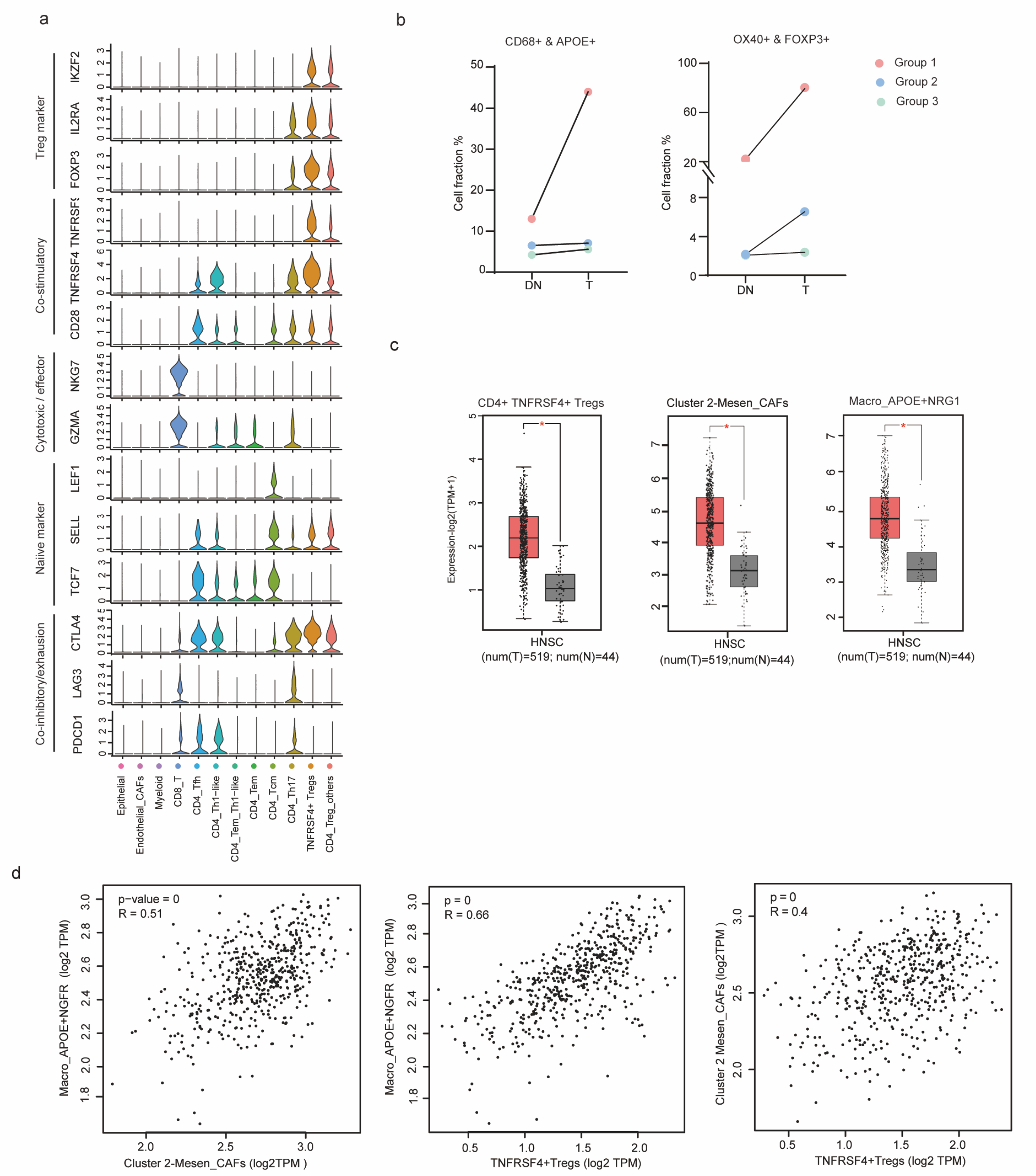

Supplementary Fig. S8

Supplementary Fig. S8. Enriched hallmark genes in TNFRSF4+Tregs and their correlations to other cell subclusters.

(a) Violin plots displaying expression levels of selected markers for inhibitory receptor, co-stimulatory molecules, naive or Treg cell markers in each T cell subpopulation. (**b**) The quantitation of CD68+ & APOE+ (left) and OX40+ & FOXP3+ (right) cell fractions in each group were provided (right). (n=3 groups). **(c)** Comparison of the expression levels of TNFRSF4+Treg, Cluster2-Mesen_CAF and

Macro_APOE/NRG1 markers between normal and tumor tissues with TCGA_HNSCC cohort data, which were done on GEPIA2 website. **(d)** Pearson Correlation maps showing the correlations between Cluster 2-Mesen_CAF and Macro_APOE/NRG1 (left); Macro_APOE/NRG1 and TNFRSF4+Tregs (middle); Cluster 2-Mesen_CAF and TNFRSF4+Tregs (right) with TCGA_HNSCC cohort data, which were done on GEPIA2 website.

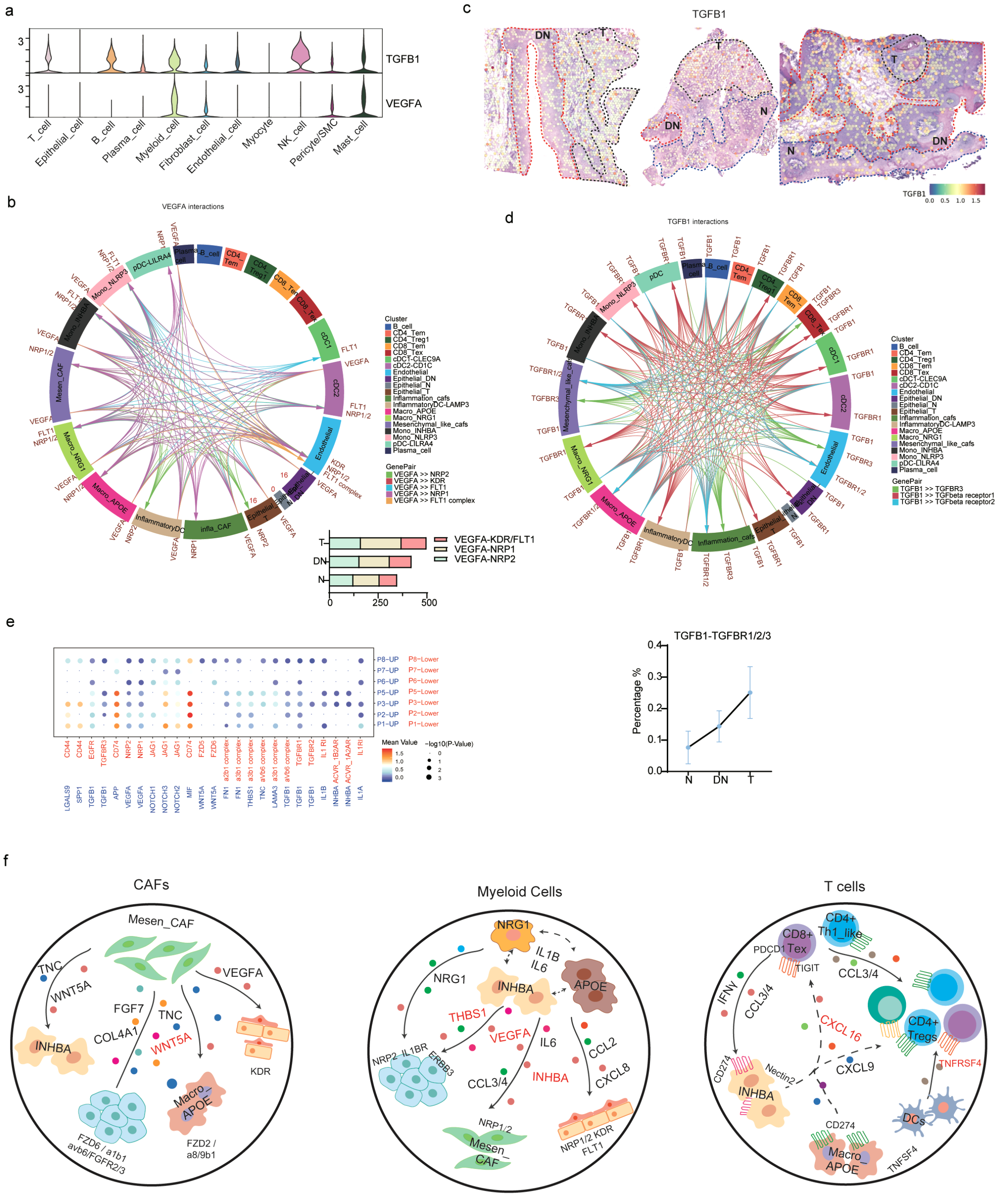

Supplementary Fig. S9

Supplementary Fig. S9. Specific cellular interactions during OSCC initiation.

(a) Violin plots of TGFB1 and VEGFA expressing levels in major cell subtypes by scRNA-seq. **(b)** Interaction maps (Top) and Statistic results (Bottom) showing VEGFA-KDR/FLT1 /NRP1/NRP2 interactions among diverse cell types. **(c)** ST feature plots showing expression of TGFB in different distributions. **(d)** Interaction maps showing TGFB-TGFBRs interactions among diverse cell types (top). Statistic results of percentage plots (bottom) among TGFB-TGFBRs in 5 representative spatial feature

maps. **(e)** Dotplots showing the significance (−log10 *P*-value) and strength (mean value) of predicted interactions between the upper layer of epithelium and lower layer of epithelium at N and DN stages. Significant mean and significance (*P*-value<0.05) were calculated based on the interaction and the normalized cell matrix achieved by Seurat Normalization. **(f)** Model of the cross-compartment ligand-receptor interactions mainly shown in terms of CAFs (left); Myeloid cells (Middle) and T cells (Right), which were supported by significant interactions inferred by CellphoneDB and the cell subclusters identified with scRNA-seq.

Supplementary Table Legends

Table S1 Patient and sample information

Table S2 Statistics for evaluating the quality of scRNA-seq (10x Genomics)

Table S3 Statistics for evaluating the quality of ST (10x Genomics)

Table S4 Statistics for cell numbers of each cluster in patients with scRNA-seq (related to Fig. 1f)

Table S5 Chosen marker genes for scoring cell type and subclusters in a cell type with a ST feature (marker genes were selected based on scRNA-seq data, related to Fig. 1g and Fig. 5e)

Table S6 Cell numbers in each cell type of different disease stages for scRNA-seq (related to Fig. 1d-f)

Table S7 Signature genes chosen for analyses on Gepia2 website (relates to Fig. S8c-d)

Table S8 Information of antibodies, primers for Real-time PCR, siRNA and reagents including chemicals, peptides and recombinant proteins
